# ERBB1 alleviates secondary brain injury induced by experimental intracerebral hemorrhage in rats by modulating neuronal death via PLC‐γ/PKC pathway

**DOI:** 10.1111/cns.14679

**Published:** 2024-03-26

**Authors:** Bing Li, Jiang Wu, Demao Cao, Cheng Cao, Juyi Zhang, Xiang Li, Haiying Li, Haitao Shen, Zhengquan Yu

**Affiliations:** ^1^ Department of Neurosurgery & Brain and Nerve Research Laboratory The First Affiliated Hospital of Soochow University Suzhou Jiangsu Province China; ^2^ Department of Neurosurgery, Yancheng City No. 1 People's Hospital, Yancheng First Hospital Affiliated Hospital of Nanjing University Medical School Yancheng Jiangsu Province China; ^3^ Department of Neurosurgery The Affiliated Hospital of Yangzhou University Yangzhou Jiangsu Province China; ^4^ Department of Neurocritical Intensive Care Unit Jiangyin Clinical College of Xuzhou Medical College Jiangyin Jiangsu Province China

**Keywords:** apoptosis, ERBB1, intracerebral hemorrhage, secondary brain injury

## Abstract

**Aims:**

Intracerebral hemorrhage (ICH) is a disease with high rates of disability and mortality. The role of epidermal growth factor receptor 1 (ERBB1) in ICH was elucidated in this study.

**Methods:**

ICH model was constructed by injecting autologous arterial blood into the right basal ganglia. The protein level of ERBB1 was detected by western blot analysis. To up‐ and downregulation of ERBB1 in rats, intraventricular injection of a lentivirus overexpression vector of ERBB1 and AG1478 (a specific inhibitor of ERBB1) was used. The cell apoptosis, neuronal loss, and pro‐inflammatory cytokines were assessed by TUNEL, Nissl staining, and ELISA. Meanwhile, behavioral cognitive impairment of ICH rats was evaluated after ERBB1‐targeted interventions.

**Results:**

ERBB1 increased significantly in brain tissue of ICH rats. Overexpression of ERBB1 remarkably reduced cell apoptosis and neuronal loss induced by ICH, as well as pro‐inflammatory cytokines and oxidative stress. Meanwhile, the behavioral and cognitive impairment of ICH rats were alleviated after upregulation of ERBB1; however, the secondary brain injury (SBI) was aggravated by AG1478 treatment. Furthermore, the upregulation of PLC‐γ and PKC in ICH rats was reversed by AG1478 treatment.

**Conclusions:**

ERBB1 can improve SBI and has a neuroprotective effect in experimental ICH rats via PLC‐γ/PKC pathway.

## Graphical abstract

ERBB1 protein level increased significantly and its main ligand, epidermal growth factor (EGF), also increased in rats after ICH. ERBB1 can improve SBI and has a neuroprotective effect in experimental ICH rats via PLC‐γ/PKC pathway.

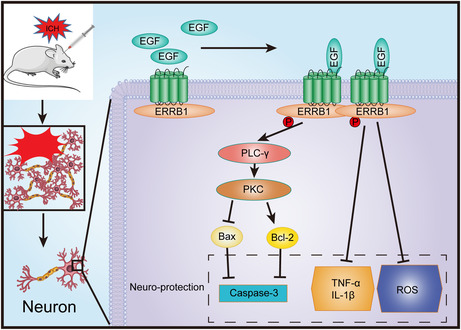

## 1 INTRODUCTION

Intracerebral hemorrhage (ICH) refers to the hemorrhage caused by non‐traumatic rupture of blood vessels in the brain parenchyma, which has an overall incidence of 24.6 per 100,000 patient years, accounting for about 15% of all stroke.^1^ Although the proportion is not high, it has a high disability rate and high mortality rate.^2^ The injury mechanism of ICH mainly includes primary brain injury, that is, the space‐occupying effect of hematoma formation, which causes mechanical compression injury to normal brain tissue, followed by secondary brain injury (SBI). The pathophysiological changes after ICH further aggravate the brain injury. The main mechanisms are activation of inflammation and induction of neuronal cell apoptosis, necrosis, and oxidative stress.^3^ With the progress of micro‐neurosurgery, patients with ICH are able to receive efficient surgical treatment, which enables the timely removal of the hematoma and a concurrent reduction in the mechanical damage caused by the hematoma's space‐occupying effect. However, such progress has not increased the proportion of patients with good prognosis; timely neurosurgical intervention only reduces the short‐term mortality of ICH, but it does not improve the long‐term prognosis of patients with ICH.^4^ Therefore, how to reduce the never injury caused by ICH is critical for improving overall patient prognosis.^5^

It has been widely recognized that cell death plays an important role in SBI after ICH, and compared with cell necrosis, apoptosis has received the most attention from researchers. The role of apoptosis was most clearly demonstrated in SBI after ICH, although its mechanisms have not been fully illustrated. It has been confirmed that EGF played an important role for post‐stroke neuronal repair, for which EGF can stimulate endogenous neural progenitor cells in the subventricular zone^6^ and contribute to nerve repair and regeneration by inhibiting apoptosis after cerebral ischemia and subarachnoid hemorrhage (SAH). Another information caught our attention is, as a corresponding receptor of EGF, the epidermal growth factor receptor (ERBB) that has also been conformed to play an important role in this process.^7^, ^8^, ^9^

The ERBB family comprises four distinct tyrosine kinase receptors, ERBB1, ERBB2, ERBB3, and ERBB4, which trigger intracellular signals in essential cellular functions including survival, differentiation, and proliferation.^10^ Previous researches on ERBB have focused largely on the development and progression of tumors, such as breast cancer, stomach cancer, and liver cancer.^11^ Recently, some studies have also begun to focus on their roles in the central nervous system.^12^ Neuregulins (NRGs), a family of proteins containing epidermal growth factor (EGF)‐like motifs, activate membrane‐associated tyrosine kinases associated with ERBBs, which has been found to regulate differentiation and migration, as well as survival of satellite cells, Schwann cells, and oligodendrocytes. In neurons, ERBB promotes neuronal migration and selectively increases the expression of other neurotransmitter receptors.^13^ Recent explorations have also found that a complex NRGs/ERBB signaling network regulates neural circuitry assembly, myelin formation, neurotransmission, and synaptic plasticity. There was an evidence, which showed that ERBB signals exist at an optimal level in the brain, and deviations from this level can impair brain function.^14^ Abnormal changes in the ERBB pathway in the brain are involved in the development of depression in chronic stress diseases, such as depression.^15^

However, the influence of ERBB family on ICH has not been reported. Therefore, the relation between ERBB1 and ICH would be investigated in this research, particularly the role of the activity of ERBB1 (regulated by phosphorylation) in SBI and the effect of ERBB1 on neuronal apoptosis following ICH. We will explore the changes of protein levels of ERBB1 and its phosphorylation levels in brain tissues, and potential roles of ERBB1 in SBI and neuronal apoptosis in an in vivo model of ICH. Additionally, it has been confirmed that the PLC‐γ/PKC pathway as downstream signals linked to ERBB1,^16^ we also explore whether the PLC‐γ/PKC pathway is involved in ERBB1‐imdiating neuroprotective role in ICH as its underlying mechanism.

## 2 MATERIALS AND METHODS

### 2.1 Ethics and Animals

All procedures involving rats were approved by the Ethics Committee of the First Affiliated Hospital of Soochow University (SDFYY‐2020368). No experimental animals were subjected to unnecessary suffering, and we used the minimum number of rats to complete all experiments. We endeavored as much as possible to reduce the suffering of animals during experiments, such as adequate anesthesia by the toe‐pinch response to pain, heating pad placement, and operations by skilled researchers, who were blinded to the groups. Male Sprague–Dawley (SD) rats (280–320 g) were purchased from the Animal Center of Chinese Academy of Sciences (Shanghai, China) and were maintained at the temperature of 23 ± 1°C on a 12‐h light/dark cycle, with relative humidity of 40% and with free access to food and water.

### 2.2 Establishment of the experimental ICH model in rats

In this study, the experimental ICH model was established in SD rats by basal ganglia injection of autologous blood.^17^ For details, please see Supplementary Materials.

### 2.3 Experimental design

This study was divided into four experiments. Before implementing the ICH model, all rats were randomly numbered and grouped by an experimenter who was not involved in the study design. Rats with earmark numbers are randomly assigned to different groups by a random‐number table, which was generated by Microsoft Excel 2019. Details of mortalities of rats in each experiment are presented in Table S1.

In Experiment 1, the change in protein levels of the ERBB family after ICH was detected. We designed Sham and ICH model groups at 3, 6, 12, 24, 48, 72, and 168 h after ICH. A total of 48 survived rats (6 rats in Sham group, and 52 rats in total in ICH groups were used, 42 of which survived after the operation) were randomly divided into eight groups of six rats each. When the precise time points were reached, the rats were sacrificed; for each ICH rat, two brain coronal sections were, respectively, collected: 3 mm before and 4 mm after coronal injection point. The former was stored at −80°C for subsequent western blot analysis and the latter was fixed with 4% paraformaldehyde at 4°C for subsequent immunofluorescence analysis (Figure S1B).

According to the basis of Experiment 1, we designed Experiment 2 to explore the role of ERBB1 in cell apoptosis, oxidative stress, and neuroinflammation in rats following experimental ICH. Experiment 2 comprised of the following six groups (six rats in each group): Sham group, ICH group, ICH + Vehicle group, ICH + AG1478 group, ICH + Vector group, and ICH + ERBB1 overexpression (OE‐ERBB1) group. At 24 h after ICH induction, they were anesthetized and two brain coronal sections in each rat were collected as described above. These samples were used for western blot analysis, ROS (reactive oxygen species) assay, TUNEL staining, and Nissl staining, respectively. Additionally, the serum and cerebrospinal fluid (CSF) of each rat were collected before anesthetized for ELISA and LDH (lactate dehydrogenase) assay (Figure S1C).

In Experiment 3, the group information was same as Experiment 2. We used 10 rats in each group for neurobehavioral scores, rotarod test, adhesive removal test, and Morris water maze test to assess the role of ERBB1 in long‐term and short‐term neurological impairment of ICH rats after various interventions (Figure S1D).

In Experiment 4, the group information was same as Experiment 2. We explored the potential molecular mechanism of ERBB1 involvement in neuroprotection in rats following experimental ICH. To save the number of rats used, the samples of Experiment 4 and Experiment 2 were shared (Figure S1E).

### 2.4 Antibodies and drugs

For details, please see Supplementary Materials.

### 2.5 Transduction of lentivirus

For overexpression of ERBB1, one ERBB1 cDNA (Gene ID:24329) was designed and cloned into the GV492 lentivirus vectors (GeneChem, Shanghai, China). Rats were anesthetized with 3% isoflurane using small animal gas anesthesia machine (R500IP; RWD Life Technology Co., Shenzhen, China), and anesthesia was maintained with 1.5% isoflurane, and then placed in a stereotaxic apparatus. A small burr hole was drilled in the skull with the coordinates of 1.5 mm posteriorly and 1.0 mm laterally relative to the bregma. A 10‐μL microliter syringe (Hamilton, Reno, NV, USA) was mounted on the frame, and the needle was inserted 3.5 mm below the bregma into the right lateral ventricle for the further injection of the lentivirus vectors. 5 μL of LV‐ERBB1‐GFP (1 × 10^9^ TU/mL) was injected for ERBB1 overexpression in brain of ICH rats, and 5 μL of LV‐Con‐GFP (4 × 10^8^ TU/mL) was used as a negative control. All lentivirus vectors were injected at a rate of 0.2 μL/min. Five minutes after injection, the needle was slowly removed. The burr hole was filled with bone wax, and the incision was gently sutured. All injections were performed 7 days before ICH induction.

### 2.6 Western blot analysis

Western blot analysis was performed according to a previous study.^18^ For details, please see Supplementary Materials.

### 2.7 Immunofluorescent analysis

Immunofluorescent analysis was performed according to a previous study.^19^ For details, please see Supplementary Materials.

### 2.8 TUNEL staining

TUNEL staining was performed according to a previous study.^20^ For details, please see Supplementary Materials.

### 2.9 Nissl staining

Nissl staining was performed according to our previous study.^21^ For details, please see Supplementary Materials.

### 2.10 ELISA

The concentrations of TNF‐α and IL‐1β in serum and CSF samples were measured using specific ELISA kits (Bio‐Swamp, China) according to the manufacturer's instructions.

### 2.11 Lactate dehydrogenase assay

The detection of LDH in CSF was performed using an LDH assay kit (Jian Cheng Biotech, China) to assess the extent of brain damage after ICH.

### 2.12 ROS assay

Based on a previous study,^22^ the ROS level was detected using a BBoxiProbe® Tissue ROS Detection Kit (BB‐470512, BestBio, Shanghai, China) according to the manufacturer's instructions. An equal amount of brain tissue surrounding the hematoma (50 mg) for each rat was used for the experiment. After homogenization with homogenization buffer A (500 μL for each sample), the homogenate was centrifuged at 100*g* for 5 min at 4°C. Next, the supernatant (190 μL) was mixed with the fluorescent probe BBoxiProbe 013 (10 μL) in black 96‐well plates for incubation for 30 min at 37°C. A fluorescence microplate reader was used to measure the fluorescence intensity of each sample.

### 2.13 Neurobehavioral scores

At 48 h after ICH, modified Garcia scoring, a sensorimotor assessment method, was used to measure neurological impairments.^23^ For details, please see Supplementary Materials.

### 2.14 Rotarod test

The rotarod test is widely used to evaluate rodent motor coordination.^24^ For details, please see Supplementary Materials.

### 2.15 Adhesive removal test

The adhesive removal test was usually used to assess sensorimotor deficits after ICH.^25^ For details, please see Supplementary Materials.

### 2.16 Morris water maze test

The Morris water maze test was performed to assess spatial learning and memory abilities in rats.^26^ For details, please see Supplementary Materials.

### 2.17 Statistical analysis

SPSS 26.0 (IBM SPSS Statistics, RRID: SCR_019096) was used for statistical analysis. All data were reported as mean ± SEM. Prior to statistical analyses, data sets were tested for normality of distribution using the Kolmogorov–Smirnov test, and the data groups (two groups) with normal distribution are compared by using two‐sided unpaired Student's *t* test, and the Mann–Whitney *U* test was used for non‐parametric data. Significant differences between groups (three groups and more) were assessed by one‐way or two‐way ANOVA. *p* < 0.05 was deemed statistically significant and all power analysis >0.8 in this study. The number of treated animals indicated by “N” in differential groups. Details of statistical analysis in each experiment are presented in Table S2.

## 3 RESULTS

### 3.1 General observation

No rats died in Sham group (0/22 rats) and, collectively, 27 rats died in ICH groups, and the mortality rate of rats in ICH groups was 18.88% (27/143 rats). There was stable hematoma volume located in the ipsilateral basal ganglia region of rats at 24 h after ICH. Representative images of Sham and ICH rats are shown in Figure S1A.

### 3.2 Changes in ERBB family protein levels after ICH

**FIGURE 1:**
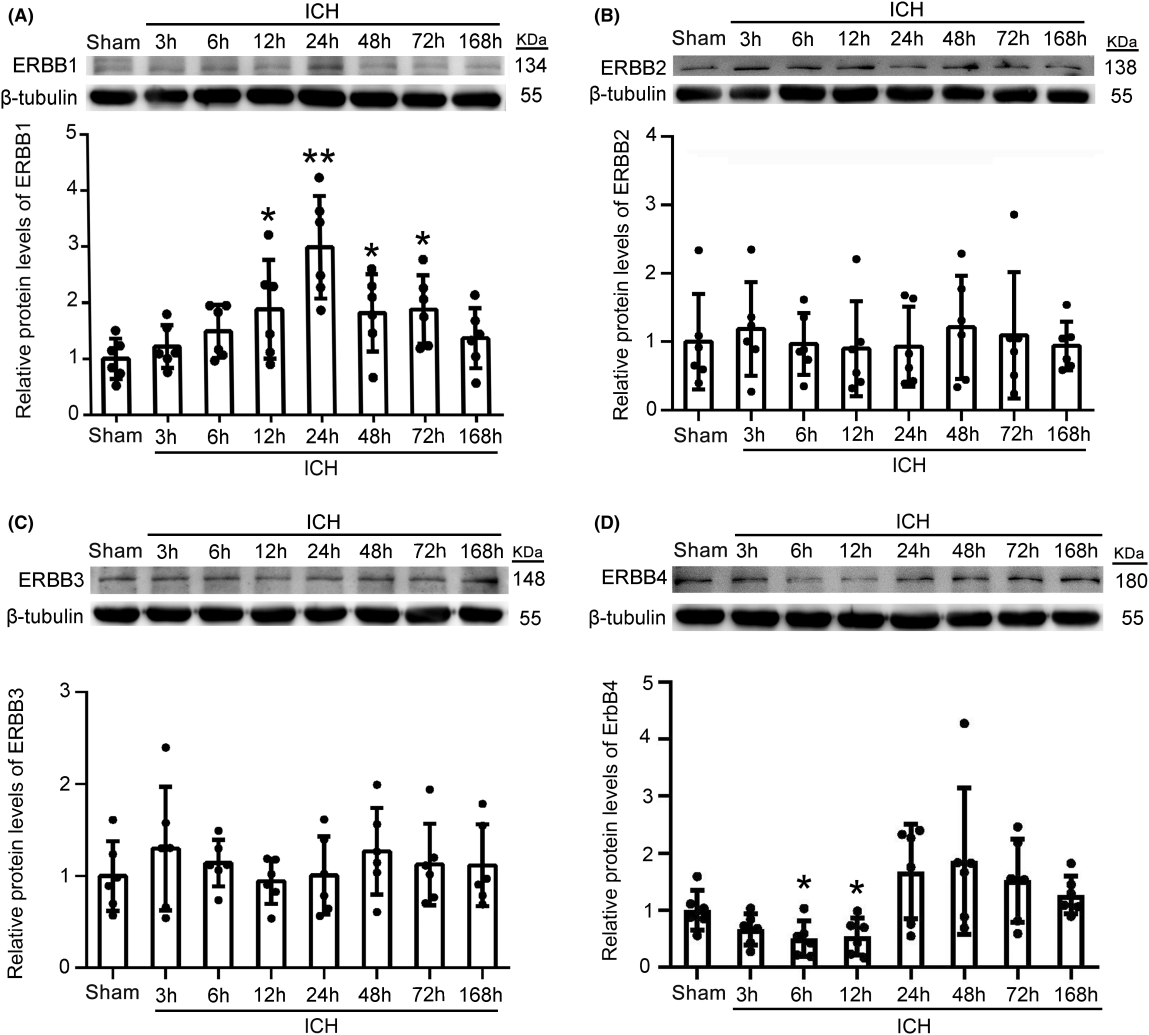
ERBB family protein levels in brain tissue around the hematoma after ICH. (A) Western blot analysis and quantification of ERBB1 levels at 3, 6, 12, 24, 48, 72, and 168 h after ICH; **p < 0.01 vs. Sham group, *p < 0.05 vs. Sham group, *n* = 6. (B–D) Western blot analysis and quantification of ERBB2, ERBB3, and ERBB4 at the above time points; *p < 0.05 vs. Sham group. All data are displayed as means ± SEM, and mean values for Sham group are normalized to 1.0; *n* = 6, the number of treated animals indicated by “N” in differential experiments.

To explore the changes of ERBB family protein levels in brain tissue of rats after ICH, the western blot analysis was conducted. Western blot analysis of total protein samples isolated from brain tissue surrounding the hematoma of ICH rats showed that, compared to those of Sham group, ERBB1 protein levels gradually increased after ICH, peaked at 24 h, and then slowly decreased (Figure 1A). However, ERBB2 and ERBB3 protein levels did not change significantly at the different time points after ICH (Figure 1B,C). Compared with Sham group, ERBB4 protein levels decreased at 6 and 12 h after ICH, but subsequently increased (Figure 1D). These results suggested that the changes in ERBB1 level were the most pronounced and were likely play an important role in brain injury after ICH. We conducted further experiments to explore the changes in protein levels of EGF, the main ligand of ERBB1, after ICH. The results showed that EGF protein levels increased significantly after ICH and reached the highest level at 12 h after ICH, which was maintained until 72 h after ICH (Figure S2A,B). The results of immunofluorescence co‐staining of NeuN (a bio‐marker of neurons) showed that the increased EGF protein levels mainly occurred in neurons (Figure S2C,D).

### 3.3 Upregulation of ERBB1 alleviated nerve injury induced by ICH

**FIGURE 2:**
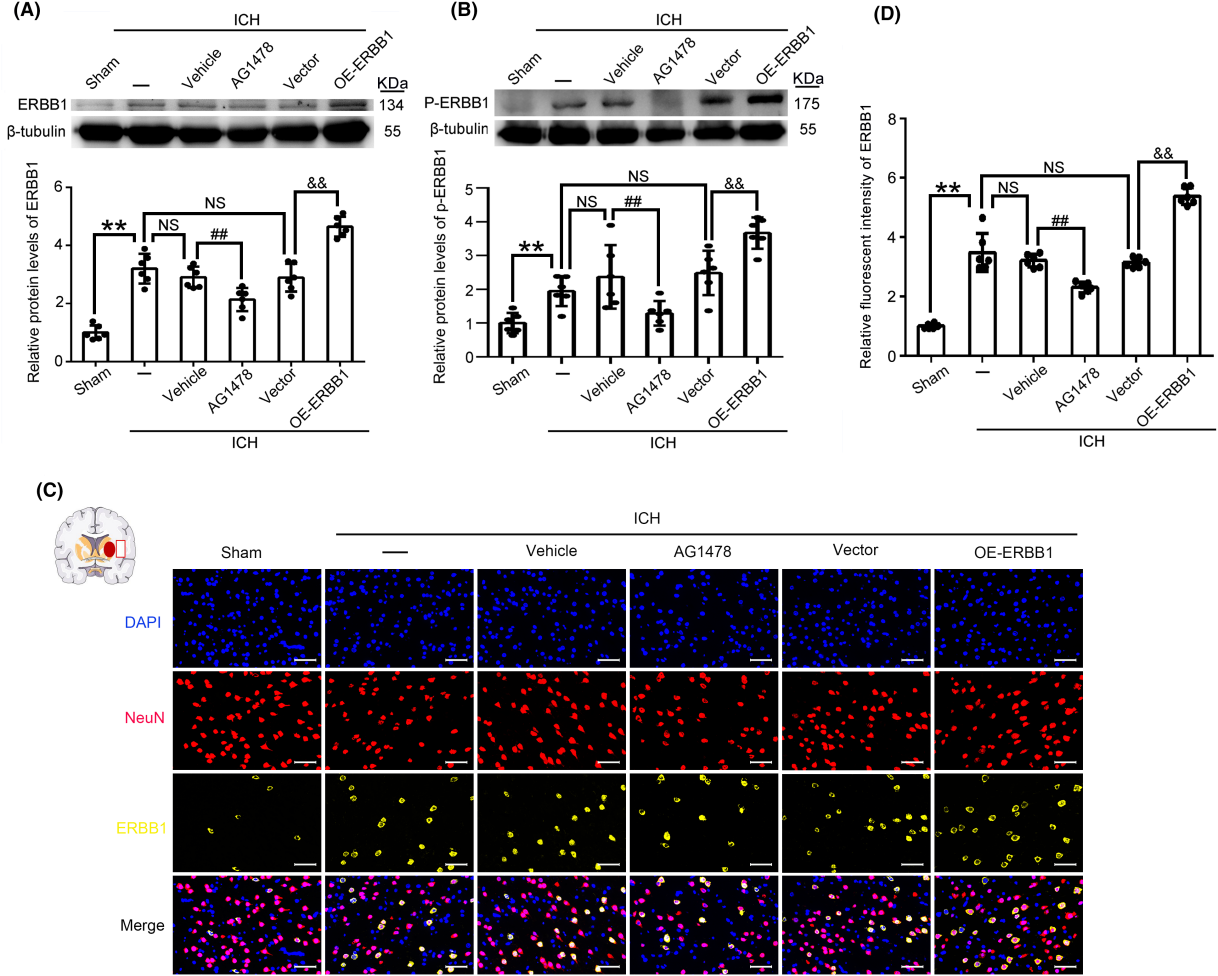
Effect of AG‐1478 treatment and the overexpression lentivirus transfection on ERBB1 levels in brain tissue of ICH rats. (A, B) Western blot analysis and quantitative analysis of ERBB1 and p‐ERBB1 levels in the different intervention groups included the specific inhibitor of ERBB1, AG‐1478 treatment group, and its vehicle group, as well as the lentivirus‐ERBB1 overexpression group and its negative control vector group; **p < 0.01 vs. Sham group; ^##^ *p* < 0.01 vs. ICH + Vehicle group; ^&&^ *p* < 0.01 vs. ICH + Vector group; NS, no significant difference vs. ICH group. (C, D) Double immunofluorescence staining on brain sections of rats in various groups was performed with ERBB1 antibody (yellow) and NeuN antibody (red). Nuclei were fluorescently labeled with DAPI (blue). Scale bar = 50 μm; **p < 0.01 vs. Sham group; ^##^ *p* < 0.01 vs. ICH + Vehicle group; ^&&^ *p* < 0.01 vs. ICH + Vector group; NS, no significant difference vs. ICH group; All data are displayed as means ± SEM, and mean values for Sham group are normalized to 1.0; *n* = 6, the number of treated animals indicated by “N” in differential experiments.

To further explore the role of ERBB1 in ICH‐induced nerve injury, we used AG‐1478 to interfere with its expression and function. Also, we constructed an ERBB1‐overexpression lentivirus vector. We firstly verified the effectiveness of both AG‐1478 and the ERBB1‐overexpression lentivirus vector treatments in ICH rats by western blot analysis. Results indicated that AG‐1478 significantly reduced ERBB1 protein level after ICH. Concurrently, the overexpression lentivirus significantly increased ERBB1 protein level (Figure 2A, Figure S3). Based on previous report,^27^, ^28^ phosphorylated ERBB1 (p‐ERBB1) is an important form of its function, and thus, we also tested the level of p‐ERBB1 in various groups. With the treatment of AG‐1478, the level of p‐ERBB1 was decreased significantly; on the contrary, with the overexpression of ERBB1 in rat brain tissues, the level of p‐ERBB1 was increased at 24 h after ICH modeling (Figure 2B). Next, immunofluorescence staining was performed to further verify the effectiveness of AG‐1478 and the overexpression lentivirus in protein levels of ERBB1 in neurons of brain tissue of rats in each group. The results suggested that ERBB1 level at 24 h post‐ICH exhibited significantly higher levels compared to the Sham group. Additionally, it also confirmed that ERBB1 was primarily expressed in neuronal cells (NeuN+ cells). AG‐1478 treatment decreased, while the overexpression lentivirus transfection increased ERBB1 protein levels in neuronal cells (NeuN+ cells) after ICH (Figure 2C,D).

**FIGURE 3:**
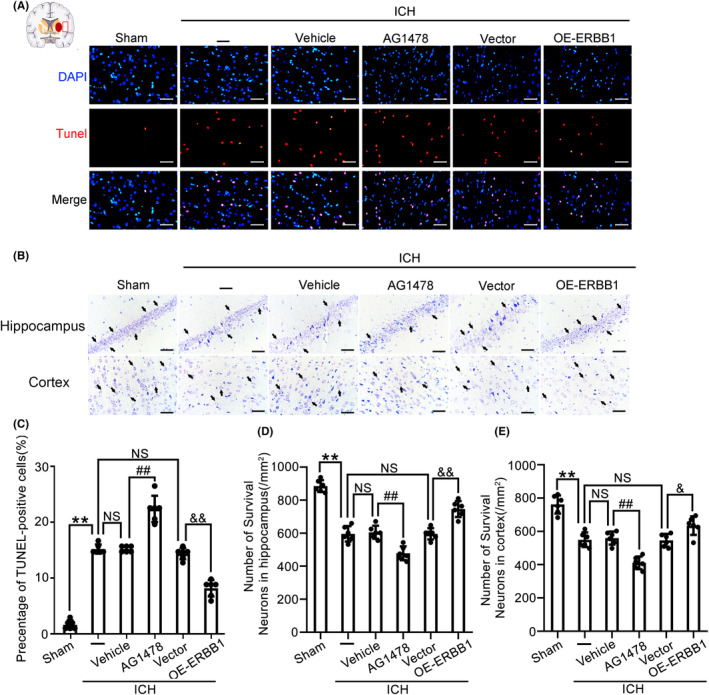
Effects of ERBB1 on cell apoptosis and neuronal loss in brain tissue of rats in various groups. (A, C) Apoptosis in brain tissues was detected by TUNEL staining after ICH. TUNEL‐positive cells were labeled in red fluorescence, and the nuclei were labeled with DAPI (blue). The percentage of TUNEL‐positive cells was statistically quantified and analyzed. Scale bar = 50 μm; **p < 0.01 vs. Sham group; ^##^ *p* < 0.01 vs. ICH + Vehicle group; ^&&^ *p* < 0.01 vs. ICH + Vector group; NS, no significant difference vs. ICH group. (B, D, E) Nissl staining was performed to measure neuronal survival in brain tissue of rats in each group. Neurons with weak staining and plump cell bodies were considered to be viable neurons. Neuronal survival in the temporal cortex near the blood clot and hippocampal region was separately observed and analyzed. Scale bar = 50 μm; **p < 0.01 vs. Sham group, ^##^ *p* < 0.01 vs. ICH + Vehicle group; ^&&^ *p* < 0.01 vs. ICH + Vector group; NS, no significant difference vs. ICH group. All data are displayed as means ± SEM; *n* = 6, the number of treated animals indicated by “N” in differential experiments.

After ERBB1 intervention, we conducted TUNEL and Nissl stainings to assess the influence of ERBB1 on cell apoptosis and neuronal loss, respectively. Specifically, compared with Sham group, TUNEL‐positive cells were significantly increased in brain tissue of rats in ICH group; in ICH + AG‐1478 group, cell apoptosis in brain tissue was increased obviously compared with ICH + Vehicle group; however, in ICH + OE‐ERBB1 group, cell apoptosis was reduced compared with ICH + Vector group (Figure 3A,C). These results suggested that upregulation of ERBB1 can reduce cell apoptosis in brain tissue induced by ICH modeling. Furthermore, the results of Nissl staining in the hippocampal CA1 region and cortex showed that the neuronal survival in ICH + AG‐1478 group was significantly decreased compared with that in ICH + Vehicle group. In contrast, in ICH + OE‐ERBB1 group, neuronal survival in the hippocampal CA1 region and cortex was increased compared with ICH + Vector group (Figure 3B–E). These results suggested that ERBB1 plays an important role in neuroprotection in brain injury caused by ICH.

**FIGURE 4:**
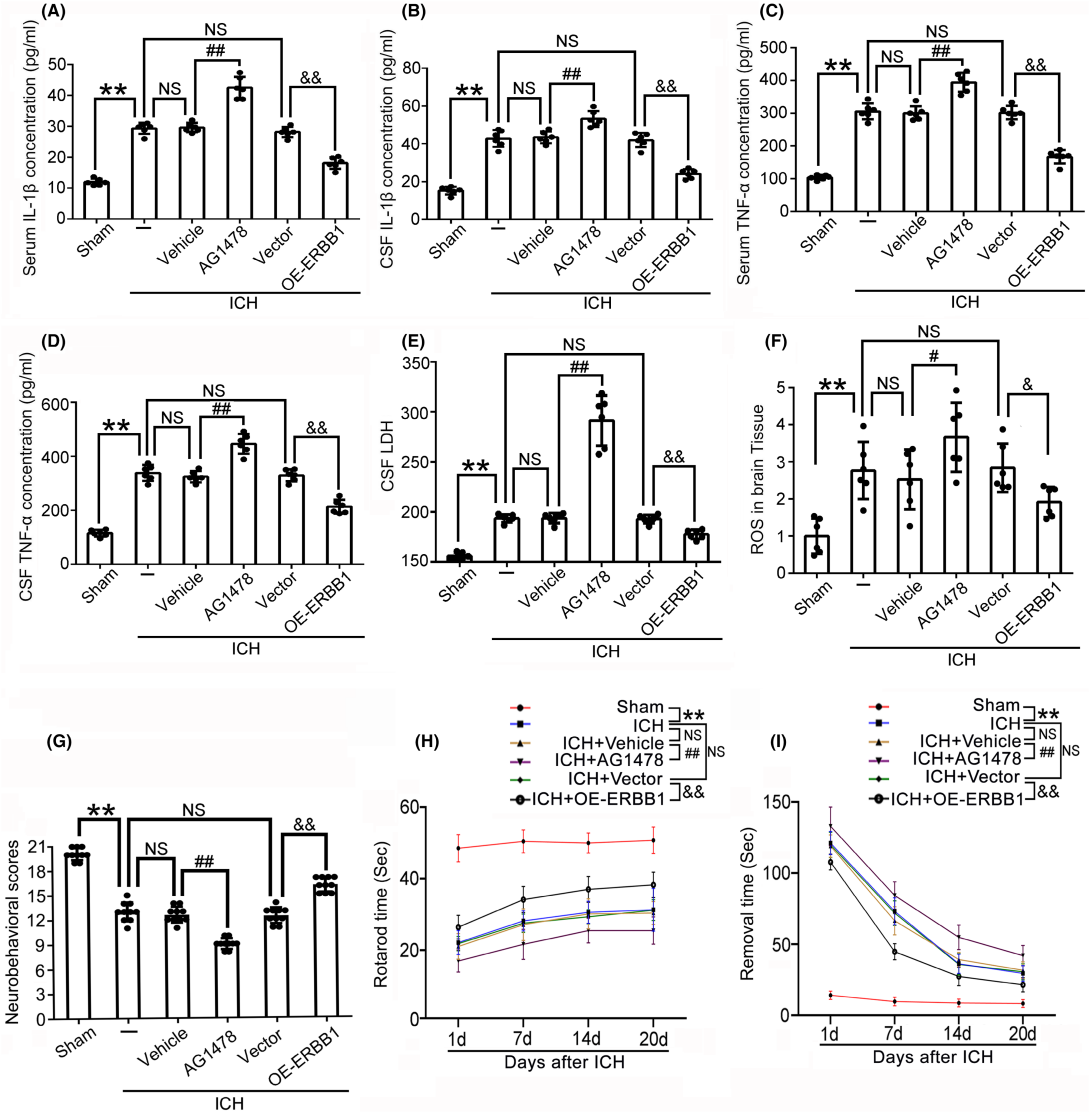
Effects of ERBB1 on pro‐inflammatory cytokines TNF‐α and IL‐1β levels, necrosis, and oxidative stress in brain tissue of rats in various groups. (A, C) The concentrations of TNF‐α and IL‐1β in serum were measured by specific ELISA kits to assess the levels of inflammation; **p < 0.01 vs. Sham group, ^##^ *p* < 0.01 vs. ICH + Vehicle group; ^&&^ *p* < 0.01 vs. ICH + Vector group; NS, no significant difference vs. ICH group. *n* = 6. (B, D) The concentrations of TNF‐α and IL‐1β in CSF were measured by the specific ELISA kits to assess the levels of inflammation in the CNS of rats; **p < 0.01 vs. Sham group, ^##^ *p* < 0.01 vs. ICH + Vehicle group; ^&&^ *p* < 0.01 vs. ICH + Vector group; NS, no significant difference vs. ICH group. *n* = 6. (E) The concentration of LDH in CSF was detected using an LDH assay kit to assess cell necrosis in the brain tissue of rats; **p < 0.01 vs. Sham group, ^##^ *p* < 0.01 vs. ICH + Vehicle group; ^&&^ *p* < 0.01 vs. ICH + Vector group; NS, no significant difference vs. ICH group. *n* = 6. (F) The ROS levels in brain tissue of rats were detected using a ROS assay kit to assess oxidative stress; **p < 0.01 vs. Sham group, ^#^ *p* < 0.05 vs. ICH + Vehicle group; ^&^ *p* < 0.05 vs. ICH + Vector group; NS, no significant difference vs. ICH group. *n* = 6. (G) The modified Garcia score was used to measure sensorimotor impairments of rats in various groups; **p < 0.01 vs. Sham group, ^##^ *p* < 0.01 vs. ICH + Vehicle group; ^&&^ *p* < 0.01 vs. ICH + Vector group; NS, no significant difference vs. ICH group. *n* = 10. (H) The rotarod test was used to evaluate the motor coordination of rats in various groups; **p < 0.01 vs. Sham group, ^##^ *p* < 0.01 vs. ICH + Vehicle group; ^&&^ *p* < 0.01 vs. ICH + Vector group; NS, no significant difference vs. ICH group. *n* = 10. (I) The adhesive removal test was used to assess sensory and coordinated movement of the left forelimb of rats in various groups; **p < 0.01 vs. Sham group, ^##^ *p* < 0.01 vs. ICH + Vehicle group; ^&&^ *p* < 0.01 vs. ICH + Vector group; NS, no significant difference vs. ICH group. *n* = 10. All data are displayed as means ± SEM, and the number of treated animals indicated by “N” in differential experiments.

We further evaluated the effect of ERBB1 on pro‐inflammatory cytokines TNF‐α and IL‐1β levels in serum and CSF, respectively. The results showed that, compared with ICH + Vehicle group, the TNF‐α and IL‐β levels in serum and CSF in ICH + AG‐1478 group were increased, which suggested that inhibiting ERBB1 by specific chemical inhibitor can aggravate the inflammatory response induced by ICH. In contrast, in ICH + OE‐ERBB1 group, the TNF‐α and IL‐β levels in serum and CSF were reduced compared with ICH + Vector group, which suggested that ERBB1 overexpression alleviated the inflammatory response after ICH (Figure 4A–D). Furthermore, we detected the level of LDH in CSF to evaluate the role of ERBB1 in the cell necrosis in brain tissue of rats in each group. The results indicated that AG‐1478 treatment aggravated, while the overexpressed ERBB1 alleviated, cell necrosis in brain tissue induced by ICH (Figure 4E). Next, we detected the ROS levels in brain tissue of rats to determine whether ERBB1 was involved in oxidative stress after ICH. The ROS levels were significantly increased after ICH compared with Sham group. In ICH + OE‐ERBB1 group, the ROS levels were reduced remarkably in brain tissue compared with ICH + Vector group. However, in ICH + AG‐1478 group, the ROS levels in brain tissue were obviously higher than that in ICH + Vehicle group, which suggested that inhibiting ERBB1 can aggravate oxidative stress in ICH‐induced brain injury (Figure 4F).

### 3.4 Overexpression of ERBB1 reduced neurological functional deficits caused by ICH

To further explore the effect of ERBB1 on neurological dysfunction of ICH rats, we employed the modified Garcia score system in this study. Compared with Sham group, rats suffered severe neurological dysfunction after ICH modeling, and in ICH + AG‐1478 group, neurological dysfunction was obviously worse; however, the behavioral scores of rats in ICH + OE‐ERBB1 group were obviously higher than those in ICH + Vector group (Figure 4G). Results of the rotarod test indicated that the rotarod time of rats after ICH was significantly shortened. Overexpression of ERBB1 in rats increased the time spent on the rotarod; however, AG‐1478 administration reduced the time on the rotarod (Figure 4H). We also conducted the adhesive removal test. After ICH, the time taken for the rats to remove the sticker was significantly longer, and the removal time further increased after administration of AG‐1478. In contrast, after administration of the overexpression lentivirus, the time to remove the stickers was less than that in ICH + Vector group (Figure 4I). Overall, these results indicated that overexpressed ERBB1 reduced behavioral damage caused by ICH.

**FIGURE 5:**
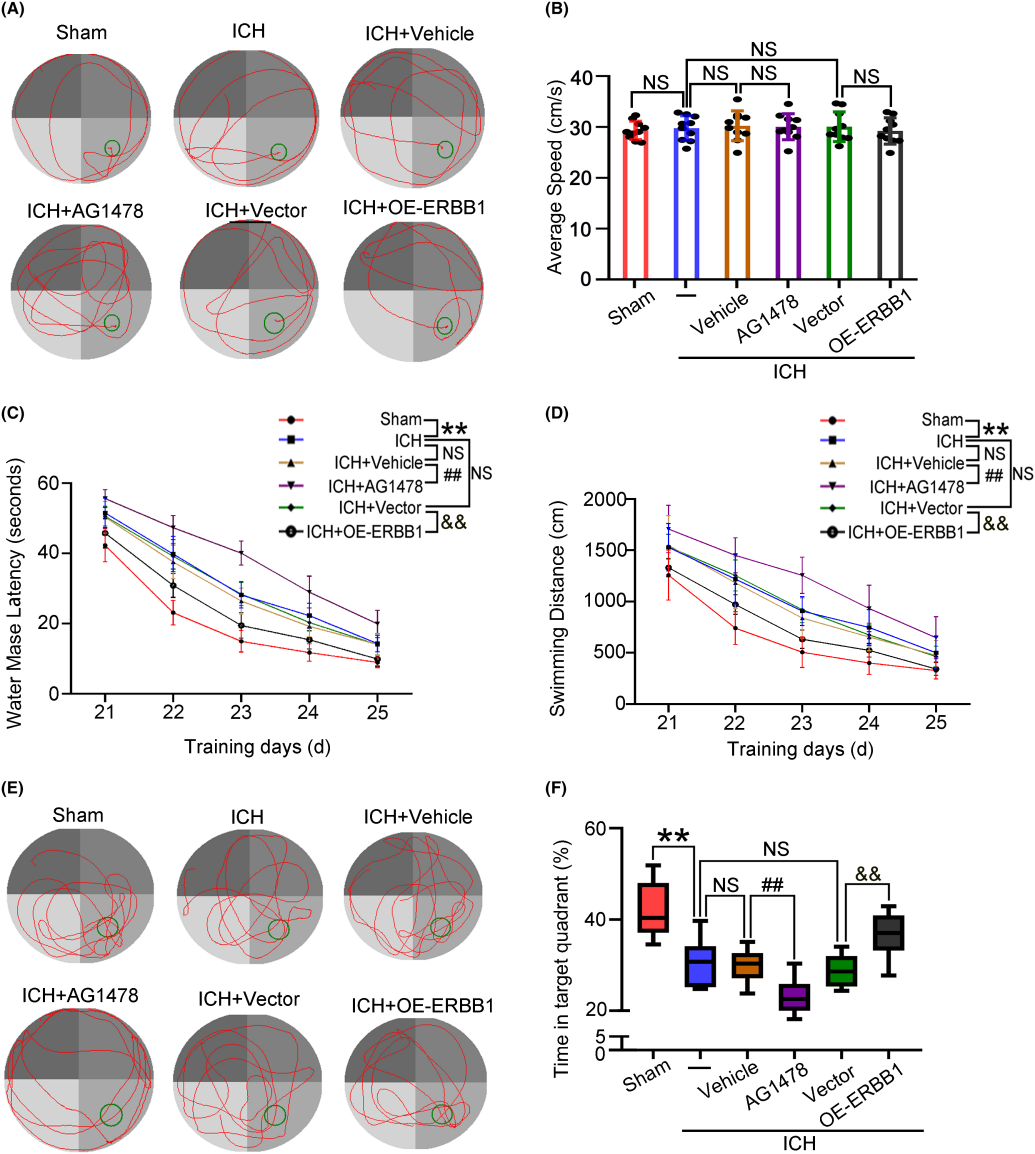
Effects of ERBB1 on spatial learning and memory of rats after ICH. (A) The typical swim path of rats in the Morris water maze test. The red line is the movement track and the green circle is the terminal platform. (B) The average swimming speed of rats in each group. NS, no significant difference, *n* = 10. (C) The time to reach the hidden platform in the Morris water maze test; **p < 0.01 vs. Sham group, ^##^ *p* < 0.01 vs. ICH + Vehicle group; ^&&^ *p* < 0.01 vs. ICH + Vector group; NS, no significant difference vs. ICH group. *n* = 10. (D) The swimming distance of each rat during the test; **p < 0.01 vs. Sham group, ^##^ *p* < 0.01 vs. ICH + Vehicle group; ^&&^ *p* < 0.01 vs. ICH + Vector group; NS, no significant difference vs. ICH group. *n* = 10. (E) The probe test in each group on day 26 after ICH modeling. The platform was removed and rats were placed at the same starting position in the pool and allowed to swim for 60s. The red line is the movement track and the green circle is the original placement of the platform. (F) The proportion of time spent in the platform quadrant; **p < 0.01 vs. Sham group, ^##^ *p* < 0.01 vs. ICH + Vehicle group; ^&&^ *p* < 0.01 vs. ICH + Vector group; NS, no significant difference vs. ICH group. *n* = 10. All data are displayed as means ± SEM, and the number of treated animals indicated by “N” in differential experiments.

The Morris water maze test was used to further determine whether ERBB1 overexpression improved long‐term cognitive functions after ICH. The rats in each group showed similar swimming speeds, indicating that the swimming ability was equivalent. Compared with the Sham group, the swimming time and distance of the rats increased after ICH. Meanwhile, after the platform was removed, the time that the rats spent in the target quadrant decreased. The results suggested that the brain damage caused by ICH resulted in long‐term learning and memory impairments. Next, compared with ICH + Vehicle group, the latency and swimming distance of rats in ICH + AG‐1478 group were longer, which suggested that spatial cognition and learning ability were more severely impaired. Also, the proportion of the time spent in the target quadrant was reduced, indicating that memory was also severely affected. However, this impairment was alleviated by overexpression of ERBB1. In ICH + OE‐ERBB1 group, the latency and swimming distance of the rats were shortened, and the proportion of time spent in the target quadrant after the platform was removed was also longer, suggesting that cognitive function was improved (Figure 5).

### 3.5 ERBB1 attenuated ICH‐induced apoptosis by activating the PLC‐γ/PKC pathway

**FIGURE 6:**
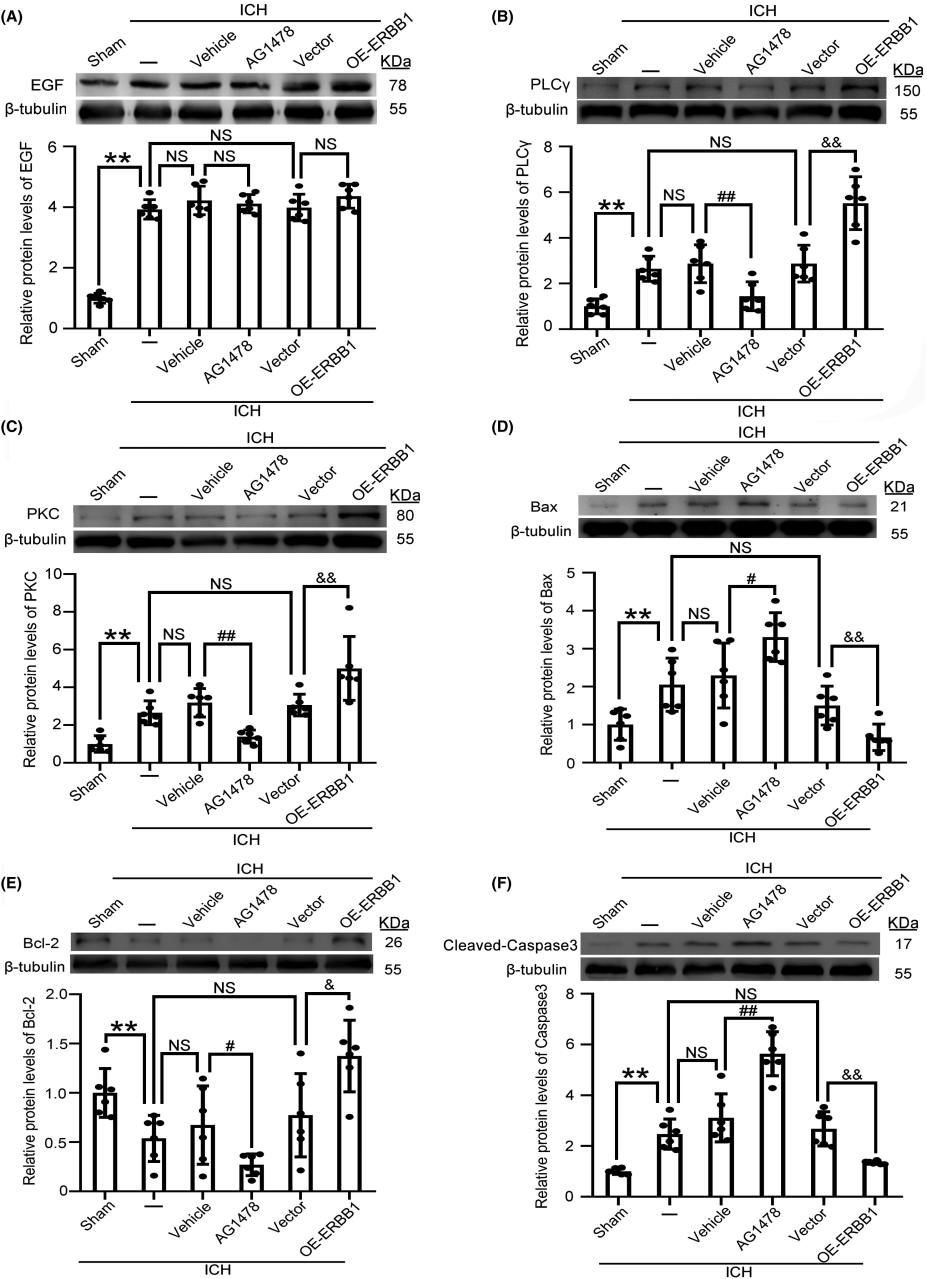
The underlying mechanism of ERBB1‐mediated neuroprotective effects after ICH. (A) Western blot analysis and quantification of EGF levels in the different intervention groups; **p < 0.01 vs. Sham group, NS, no significant difference vs. ICH + Vehicle group; NS, no significant difference vs. ICH + Vector group; NS, no significant difference vs. ICH group. (B) Western blot analysis and quantification of PLC‐γ levels in the different intervention groups; **p < 0.01 vs. Sham group, ^##^ *p* < 0.01 vs. ICH + Vehicle group; ^&&^ *p* < 0.01 vs. ICH + Vector group; NS, no significant difference vs. ICH group. (C) Western blot analysis and quantification of PKC levels in the different intervention groups; **p < 0.01 vs. Sham group, ^##^ *p* < 0.01 vs. ICH + Vehicle group; ^&&^ *p* < 0.01 vs. ICH + Vector group; NS, no significant difference vs. ICH group. (D) Western blot analysis and quantification of Bax levels in the different intervention groups; **p < 0.01 vs. Sham group, ^##^ *p* < 0.01 vs. ICH + Vehicle group; ^&&^ *p* < 0.01 vs. ICH + Vector group; NS, no significant difference vs. ICH group. (E) Western blot analysis and quantification of Bcl‐2 levels in the different intervention groups; **p < 0.01 vs. Sham group, ^##^ *p* < 0.01 vs. ICH + Vehicle group; ^&&^ *p* < 0.01 vs. ICH + Vector group; NS, no significant difference vs. ICH group. (F) Western blot analysis and quantification of cleaved caspase‐3 levels in the different intervention groups; **p < 0.01 vs. Sham group, ^##^ *p* < 0.01 vs. ICH + Vehicle group; ^&&^ *p* < 0.01 vs. ICH + Vector group; NS, no significant difference vs. ICH group. All data are displayed as means ± SEM; *n* = 6, the number of treated animals indicated by “N” in differential experiments.

It has been confirmed that the PLC‐γ/PKC pathway is an important downstream signal linked to ERBB1.^16^ To explore whether the PLC‐γ/PKC signaling pathway was involved in ERBB1‐mediating neuroprotective role in ICH, we performed western blot analysis after precise regulation of ERBB1 to observe changes in PLC‐γ and PKC protein levels. Firstly, we noticed that ERBB1 interventions did not affect EGF protein levels (Figure 6A). Next, the protein levels of phospholipase C‐γ (PLC‐γ) and protein kinase C (PKC) increased after ICH compared with Sham group. This result suggested that the ERBB1/PLC‐γ/PKC pathway is activated after ICH. Significantly, compared with ICH + Vehicle group, the protein levels of PLC‐γ and PKC in ICH + AG‐1478 group decreased significantly, while in the OE‐ERBB1 group, the protein levels of PLC‐γ and PKC increased significantly (Figure 6B,C).

Another research also reported that phosphorylation of ERBB1 suppressed the caspase activation and apoptosis.^29^ In order to explore whether the apoptosis signaling pathway was affected by ERBB1 intervention in ICH in this study, the protein levels of anti‐apoptotic molecular Bcl‐2, pro‐apoptotic molecular Bax, and apoptotic executive protein cleaved caspase‐3 were detected.^30^ The results showed that overexpression of ERBB1 could significantly increase the level of Bcl‐2 and decrease the level of Bax, also significantly reducing cleaved caspase‐3. However, in ICH + AG‐1478 group, when ERBB1 was inhibited, the protein levels of Bcl‐2 decreased, while Bax increased, and increasing the cleaved caspase‐3 (Figure 6D–F). These above results indicated that ERBB1 inhibited apoptosis might by activating PLC‐γ/PKC pathway after ICH.

## 4 DISCUSSION

Spontaneous non‐traumatic ICH is an important cause of death and morbidity worldwide.^31^ Recent large‐scale clinical trials have shown that early intensive blood pressure reduction is a safe and feasible strategy for ICH,^32^ but recent trials have failed to prove the overall beneficial effect of surgical intervention on mortality and functional outcomes.^33^, ^34^ These reports suggested that how to reduce the secondary brain damage caused by ICH was important to improve the overall prognosis of patients with ICH. Due to the important role of apoptosis involved in SBI of ICH, anti‐apoptotic research becomes particularly attractive.

For the neuroprotective function of EGF in ischemia stroke and SAH, we speculated that it has a similar role in ICH, while our study was in progress, a study reported that injectable gelatin hydrogel containing epidermal growth factor (Gtn‐EGF) can improve neurological recovery in a collagenase injection into the striatum‐induced ICH model.^35^ To further explore the molecular mechanism of the neuroprotective function of EGF in ICH, we choose its corresponding receptor, ERBB, as research objective in this study. Firstly, the changes of ERBB family members (ERBB1/2/3/4) in brain tissue after ICH modeling were, respectively, detected. The results indicated that the levels of ERBB1 were significantly increased in brain tissue at 24 h after ICH, and immunofluorescence analyses suggested that increasing ERBB1 is mainly located in neurons in peripheral hematoma brain tissue of ICH rats. These results suggested that ERBB1 may be involved in SBI induced by ICH. We further investigate the effects of these changes in protein levels of ERBB1 on SBI. The specific chemical inhibiting induced by AG1478 and genetic overexpression of ERBB1 induced by lentivirus vector were applied, and the results showed that genetic overexpression of ERBB1 levels could alleviate the degree of SBI induced by ICH through decreasing the numbers of apoptotic cells and increasing the numbers of survival neurons in brain tissue, and reducing pro‐inflammatory cytokines TNF‐α and IL‐1β levels, and decreasing cellular necrosis, and dropping oxidative stress, and improving the neurological impairment and cognitive dysfunction in rats following ICH. However, decreasing of ERBB1 by AG1478 treatment produces the opposite effects. Collectively, these results indicated that increased ERBB1 could improve SBI and play a neuroprotective effect in pathophysiological progression of ICH.

Furthermore, we explored the molecular mechanism of neuroprotective function of ERBB1 in ICH. Previous studies have shown that the ERBB/EGFR family is involved in the primarily regulating cell growth and cell migration and blocking of apoptosis.^11^ A previous study confirmed that the PLC‐γ/PKC pathway as downstream signals linked to ERBB1.^16^ It has been also confirmed that many extracellular factors, such as cytokines and growth factors, can regulate cell metabolism through the PLC‐γ/PKC pathway. PKC is a member of the serine/threonine protein kinase family, and several subtypes of PKC are involved in the regulation of apoptosis, which is the main form of neuronal death after ICH.^36^ Thus, we detected the protein levels of PLC‐γ and PKC; in this study, the results showed that decreasing of ERBB1 by AG1478 treatment reduced the levels of PLC‐γ and PKC, while increased ERBB1 could produce the opposite effects. It suggested that the PLC‐γ/PKC signal pathway was involved in the neuroprotective function induced by ERBB1 in ICH. The activation of PLC‐γ by ERBB1 regulates multiple cellular functions. PLC‐γ contains two SH2 domains, one SH3 domain, and two pleckstrin homology (PH) domains. It catalyzes the hydrolysis of phosphatidylinositol‐4,5‐bis‐phosphate (PtdIns‐4,5‐P2), creating inositol‐1,4,5‐triphosphate (IP3) and diacylglycerol (DAG). These second messengers (IP3 and DAG) can stimulate the release of Ca^2+^ from internal stores and activate PKC. It has been summarized that PLC‐γ forms a complex with ERBB1 through its SH2 domain interaction. Complex formation leads to the phosphorylation of PLC‐γ on tyrosine residues and an increase in its enzymatic activity.^12^ It should be noted that the conclusions of these molecular mechanisms are derived from molecular biology experiments in some cell lines, and whether they still exist in the pathophysiological environment of diseases (such as ICH) needs further investigation.

We further measured three biomarkers of apoptosis (Bcl‐2, Bax, and cleaved caspase‐3); in this study, the results suggested that regulation of ERBB1 affects apoptosis, and this echoes the TUNEL experiment. Thus, we inferred that ERBB1 activates the PLC‐γ/PKC pathway and inhibits cellular apoptosis, ultimately exerting neuroprotective effect in SBI after ICH. Several reports indicating an anti‐apoptotic activity for PKC have been published. For instance, downregulation of PKC using antisense nucleotides or phorbol esters treatment leads to apoptosis in different cell types. However, the mechanism of anti‐apoptotic activity by PKC is still poorly understood. A previous study demonstrated that suppression of apoptosis in murine growth factor‐dependent cell lines involves Bcl‐2 phosphorylation at serine 70 by PKC. Phosphorylated site leads to Bcl‐2 stabilization and enhances anti‐apoptotic activity.^37^ We also noticed that a previous study reported that dihydrochloride (a PKC inhibitor) treatment can reduce neuronal death induced by ICH.^38^ The method of ICH modeling (anesthesia method and blood injection amount) is quite different from our study, which may directly lead to different severity of brain injury in rats and different effects on PKC. These suggested that it is an important direction for our future work to fully explore the role of PLC‐γ/PKC pathway for different ICH models.

Additionally, AG‐1478 is a highly selective ERBB1 inhibitor that not only inhibits ERBB1 expression but also inhibits ERBB1 self‐phosphorylation and its tyrosine kinase activity.^39^ Thus, we detected the changes in levels of phosphorylated ERBB1 in various groups, and the results showed that AG‐1478 significantly reduced ERBB1 phosphorylation (p‐ERBB1), while the overexpression lentivirus vector transfection significantly increased p‐ERBB1. These results indicated that the tyrosine kinase activity of ERBB1 may be its central role of neuroprotective function in ICH. Additionally, as mentioned above, the neuroprotective function of EGF in ischemia stroke has been reported, while the ERBB4, but not ERBB1, as the main receptor to play the neuroprotective role.^40^, ^41^ Combined these reports and our results, we inferred that although the ERBB family plays a similar protective role both in cerebral ischemia and ICH, they are performed by different members. This also suggested that there is still much to be explored for the regulatory mechanism of ERBB family proteins in different diseases.

In addition, this study has several shortcomings. First, clinically, patients with ICH are mostly elderly with basic diseases such as hypertension and hyperglycemia.^31^ In such diseased groups, the pathophysiological mechanism of ICH is often complicated and changeable; in the current study, the animals used were all young male SD rats, which do not fully simulate the clinical condition. Second, we considered that there were interactions between various cells of the nervous system in the body, and there are neuronal‐glial networks and neurovascular networks, which can better reflect the various cells in the nervous system.^42^ Third, intraventricular injection of the overexpression lentivirus was used to achieve upregulation of ERBB1. Such intervention enabled rats to obtain a relatively good neurological prognosis after ICH and reduced brain injury. However, this intervention approach is difficult to achieve in clinical application and is not as feasible as intravenous injection or oral administration. Also, this genetic intervention carries increased risks in the clinic. Fourth, several types of programmed cell death occur after ICH including necrosis, apoptosis, ferroptosis, and pyroptosis.^30^ In this study, we just focused on apoptosis after ICH and did not investigate other types of cell death. ERBB1 may also be involved in the regulation of other types of cell death, which is a direction of our future work.

Overall, our study showed that, compared with other members of the ERBB family, the levels of ERBB1 and its main ligand, EGF, increased significantly after ICH. ERBB1 upregulation reduced apoptosis and neuronal loss after ICH and mitigated inflammation, cell death, and oxidative stress. Meanwhile, the long‐term behavioral and cognitive impairment of rats were also alleviated. Meanwhile, we confirmed that ERBB1 plays a neuroprotective role in inhibiting apoptosis and might through activating the PLC‐γ/PKC pathway. Therefore, it suggested the ERBB1 may be a target for inhibiting apoptosis after ICH, and the specific mechanism of the ERBB1‐PLC‐γ/PKC pathway should be further studied.

## AUTHOR CONTRIBUTIONS

All authors contributed to the study conception and design. **Bing Li**: Conceptualization, Methodology, Investigation, Validation, Writing – Original Draft, Visualization; **Jiang Wu**: Methodology, Investigation, Validation, Writing – Original Draft, Visualization; **Demao Cao**: Methodology, Writing – Original Draft, Visualization; **Cheng Cao**: Methodology, Investigation, Validation; **Juyi Zhang**: Investigation, Validation, Formal analysis; **Xiang Li**: Methodology, Formal analysis; **Haiying Li**: Methodology, Formal analysis; **Haitao Shen**: Conceptualization, Writing – Review & Editing, Visualization, Supervision, Funding acquisition; **Zhengquan Yu**: Conceptualization, Writing – Review & Editing, Supervision.

## CONFLICT OF INTEREST STATEMENT

The authors have no relevant financial or non‐financial interests to disclose.

## Supporting information


Appendix S1

Table S1

Appendix S2

## Data Availability

The datasets generated during and/or analyzed during the current study are available from the corresponding author on reasonable request.
