# Supplementary material for: ERBB1 alleviates secondary brain injury induced by experimental intracerebral hemorrhage in rats by modulating neuronal death via PLC‐γ/PKC pathway
Source: CNS Neurosci Ther. 2024 Mar 26;30(3):e14679. doi: 10.1111/cns.14679 (PMC10964039; doi:10.1111/cns.14679)
Supplement: Supplementary file 1 — Appendix S1 [file CNS-30-e14679-s002.docx]

**ERBB1 alleviates secondary brain injury induced by experimental intracerebral hemorrhage in rats by modulating neuronal death via PLC-γ/PKC pathway**

**Running title: The role of ERBB1 in experimental ICH rats**

Bing Li^1,2#^, Jiang Wu^1#^, Demao Cao^1,3^, Cheng Cao^1,4^, Juyi Zhang^1^, Xiang Li^1^, Haiying Li^1^, Haitao Shen^1^*, Zhengquan Yu^1^*

1 Department of Neurosurgery & Brain and Nerve Research Laboratory, The First Affiliated Hospital of Soochow University, 188 Shizi Street, Suzhou 215006, Jiangsu Province, China.

2 Department of Neurosurgery, Yancheng City No.1 People's Hospital, Yancheng First Hospital, Affiliated Hospital of Nanjing University Medical School, Yancheng 224006, Jiangsu Province, China.

3 Department of Neurosurgery, The Affiliated Hospital of Yangzhou University, Yangzhou, Jiangsu Province, China.

4 Department of Neurocritical Intensive Care Unit, Jiangyin Clinical College of Xuzhou Medical College, Jiangyin, Jiangsu Province, China.

#These authors contributed equally to this work.

*Corresponding author: Haitao Shen and Zhengquan Yu, Department of Neurosurgery & Brain and Nerve Research Laboratory, The First Affiliated Hospital of Soochow University, 188 Shizi Street, Suzhou 215006, China. E-mail: dagezi120@126.com (Haitao Shen) and zhengquan_yu@126.com (Zhengquan Yu).

**Supplemental Materials and methods**

**Establishment of the experimental ICH model in rats**

In this study, the experimental ICH model was established in SD rats by basal ganglia injection of autologous blood.(17) Rats were anesthetized with 3% isoflurane using small animal gas anesthesia machine (R500IP; RWD Life Technology Co., Shenzhen, China), and anesthesia was maintained with 1.5% isoflurane, and then rats were fixed on a stereotaxic frame (Zheng Hua Biological Equipment Co. Ltd, Anhui, China) in a prone position. The hair on the head was removed, and the exposed skin were disinfected, after which a median incision of the skin was made to expose the skull. A hole was then drilled to reach the right basal ganglia (3.5mm to the right and 0.2-mm posterior to bregma), after which a 100µl of autologous blood that was collected from the tail artery was slowly injected into the right basal ganglia with a micro injector at the speed of 20µl/min, and with a puncture depth 5.5mm below the brain plane. After 5min, the microinjector was slowly removed, the skull window sealed with bone wax and the scalp sutured. In the same way, experimental rats in the Sham group were injected with 100µl physiological saline solution. Representative images of the Sham and ICH groups are shown in Figure S1A.

**Antibodies and drugs**

Rabbit polyclonal anti-ERBB1/EGFR antibody (AF6043; RRID: AB_2834971), rabbit polyclonal anti-ERBB2/HER2 antibody (AF7681; RRID: AB_2844045), rabbit polyclonal anti-ERBB3/HER3 antibody (AF7761; RRID: AB_2844125), rabbit polyclonal anti-ERBB4/HER4 antibody (AF4775; RRID: AB_2844767), rabbit polyclonal anti-EGF antibody (DF2225; RRID: AB_2839456), rabbit polyclonal anti-PLCG1/PLC-γ antibody (AF6210; RRID: AB_2835091)，rabbit polyclonal anti-PKC antibody (AF6196; RRID: AB_2835077), rabbit polyclonal anti-Bax antibody (AF0120; RRID: AB_2833304), rabbit polyclonal anti-Bcl-2 antibody (AF6139; RRID: AB_2835021) and rabbit polyclonal anti-cleaved-caspase-3 antibody (AF7022; RRID: AB_2835326) were purchased from Affinity Bioscience (China). Rabbit polyclonal anti-p-ERBB1/p-EGFR (phosphor Y1173) antibody (ab5652; RRID: AB_305020) was purchased from Abcam (USA). Rabbit polyclonal anti-β-tubulin antibody (Cat#2146) was purchased from Cell Signaling Technology (USA). Secondary antibodies anti-rabbit IgG HRP-linked antibody (sc-2004; RRID: AB_631746), for western blot analysis, was purchased from Santa Cruz Biotechnology (USA). Mouse monoclonal Anti-NeuN antibody [1B7] - Neuronal Marker (ab104224; RRID: AB_10711040) was also purchased from Abcam (USA). Secondary antibodies for immunofluorescence analysis included Alexa Fluor-488 donkey anti-rabbit IgG antibody (A21206; RRID: AB_2535792) and Alexa Fluor-555 donkey anti mouse IgG antibody (A31570; RRID: AB_2536180) were purchased from Invitrogen (USA). Additionally, for chemical intervention of ERBB1 in this study, the AG-1478 (Tyrphostin AG-1478, Cat#S2728), a selective ERBB1/EGFR inhibitor, was purchased from Selleck chemicals (China). It was dissolved in DMSO at a final concentration of 30μg/μl, and finally, intraventricular injection was performed 1h before ICH modeling according to the weight of rats at a dose of 1μg/g. (39)

**Western blot analysis**

Western blot analysis was performed according to a previous study.(18) Brain tissue surrounding the hematoma collected from ICH rats in *Experiment 2* were homogenized and lysed in lysis buffer (Beyotime, China), after which the samples were centrifuged at 12000*g* for 10min at 4°C. The supernatant was collected in a new centrifuge tube and enhanced BCA Protein Assay Kits (Beyotime, China) were used to detect the concentration of the protein in each sample. The appropriate amount of protein was loaded onto a 10% SDS polyacrylamide gel and electro-transferred onto a nitrocellulose membrane (Millipore Corporation, USA). The membrane was then blocked in 5% bovine serum albumin (BSA, BIOSHARP, China) for 1h at room temperature. Then, the membrane was incubated with the primary antibodies (details were described above) overnight at 4°C on a shaking table. Next, the membrane was incubated with an HRP-conjugated secondary antibody for 1h at room temperature and then washed with PBST (PBS and 0.1% Tween 20) three times. Finally, an enhanced chemiluminescence kit (ECL, Thermo, USA) was applied to detect band signals. All subsequent analyses were carried out by experimenters who were blind to the experimental design using Image J software (National Institutes of Health, USA).

**Immunofluorescent analysis**

Based on a previous study,(19) after perfusion with physiological saline, brain tissue was removed and fixed overnight in 4% paraformaldehyde and then embedded in paraffin, cut into brain sections (4μm thickness slice). Antigen repair was performed with citrate antigen retrieval solution, after which the sections were blocked with 5% BSA and incubated with primary antibodies overnight at 4°C. Sections were then incubated with suitable secondary antibodies for 1h at 37°C in dark. Subsequently, after washing in PBST three times, the sections were stained with DAPI (Southern Biotech, USA). Finally, the sections were observed using a fluorescent microscope (ECLIPSE Ni-U Nikon, Japan) by experimenters who were blind to the experimental design.

**TUNEL staining**

TUNEL staining was performed to investigate cell apoptosis in brain tissue after ICH using the In Situ Cell Death Detection Kit (Roche, USA) as described in a previous study.(20) Brain section preparations were performed as described in the section of “***Immunofluorescent analysis***”. Then, the sections were incubated with the TUNEL reaction mixture for 1h at 37°C and, after staining with DAPI, the TUNEL-positive cells were observed using a fluorescence microscope (ECLIPSE Ni-U, Nikon, Japan,). TUNEL-positive cells were counted by an experimenter who was blinded to the study. The ratio of TUNEL-positive cells/total cells in each section was calculated and analyzed statistically.

**Nissl staining**

Neuronal loss was measured by Nissl staining as our previous study.(21) Briefly, brain sections were incubated with 0.5% toluidine blue for 30min at 37°C, after which the sections were cover-slipped with neutral resins and observed through a light microscope (Nikon, Japan) by an experimenter who was blinded to the experimental groups. We separately observed neuronal loss in the temporal cortex near the blood clot and hippocampal region. Neurons with pale nuclei and large cellular bodies were counted as viable neurons. Deep stained neurons with shrunken cell bodies were considered dead and excluded from the Nissl counting. The data measured the number of lived neurons per mm^2^ of brain section.

**Neurobehavioral scores**

At 48h after ICH, modified Garcia scoring, a sensorimotor assessment method, was used to measure neurological impairments.(23)The scoring system consists of seven tests: (a) spontaneous activity, (b) body proprioception, (c) tentacles reaction, (d) lateral turning, (e) forelimb walking, (g) limb symmetry and (h) climbing. We scored each test from 0 to 3, and rats without neurological impairments were given a score of 21 points. Researchers were blinded to the experimental groups recorded all the data.

**Rotarod test**

The rotarod test is widely used to evaluate rodent motor coordination.(24) Briefly, rats were put on the rotarod cylinder (ZH-300B, Anhui Zheng Hua Biological Equipment Co. Ltd., China), the speed raised at a constant rate from 5 to 35 r/min at an acceleration of 0.5 r/s and the time that the animals remained on the rotarod cylinder was recorded as the ‘rotarod time’. Five days before the ICH modeling, the rats were trained to familiarize themselves with the speed and environment of the rotarod apparatus. The test was then conducted on the 1st, 7th, 14th and 20th days after ICH.

**Adhesive-removal test**

The adhesive-removal test was usually used to assess sensorimotor deficits after ICH.(25) To reduce stress, the rats were moved to a transparent glass box before being tested, and then a 9-mm circular sticker was then placed on the palm of the contralateral distal forelimb. The time taken to remove all the stickers was recorded. Before ICH, all rats were trained for three days to ensure proper removal of the sticker. The test was performed on the 1st, 7th, 14th and 20th days after ICH. An experimenter was blinded to the experimental groups performed the test.

**Morris water maze test**

The Morris water maze test was performed to assess spatial learning and memory abilities in rats.(26) We evaluated the spatial cognition and learning ability of rats by recording the time and swimming distance taken by the rats to find the hidden platform, and then after the platform was removed, we counted the proportion of time the rats spent in the target quadrant to evaluate the memory. A circular pool (180cm in diameter) with a circular platform (20cm in diameter) was used to carry out the experiment. The platform was placed about 1.5cm under the water surface in the fourth quadrant of the pool. The rat was then placed in the second quadrant and allowed to find the hidden platform within 60s. The time taken to reach and stay on the platform was taken as the latency time. The swimming distance was also recorded as an indicator for assessment. After, each rat was allowed to remain on the platform for another 30s to recall the spatial environment. From day21 to day25 after ICH, the above experiments were conducted three times per day, the mean latency time and swimming distance were recorded and the average swimming speeds were recorded and analyzed. A 60s probe test was performed on day26 after ICH, whereby the platform was removed and the rats were placed at the same starting position in the pool and allowed to swim for 60s. The experimenter recorded the time that the rat spent in the platform quadrant to assess spatial memory. All behavioral tests were performed by experimenters who were blinded to the experimental groups.

**Supplemental Figures**


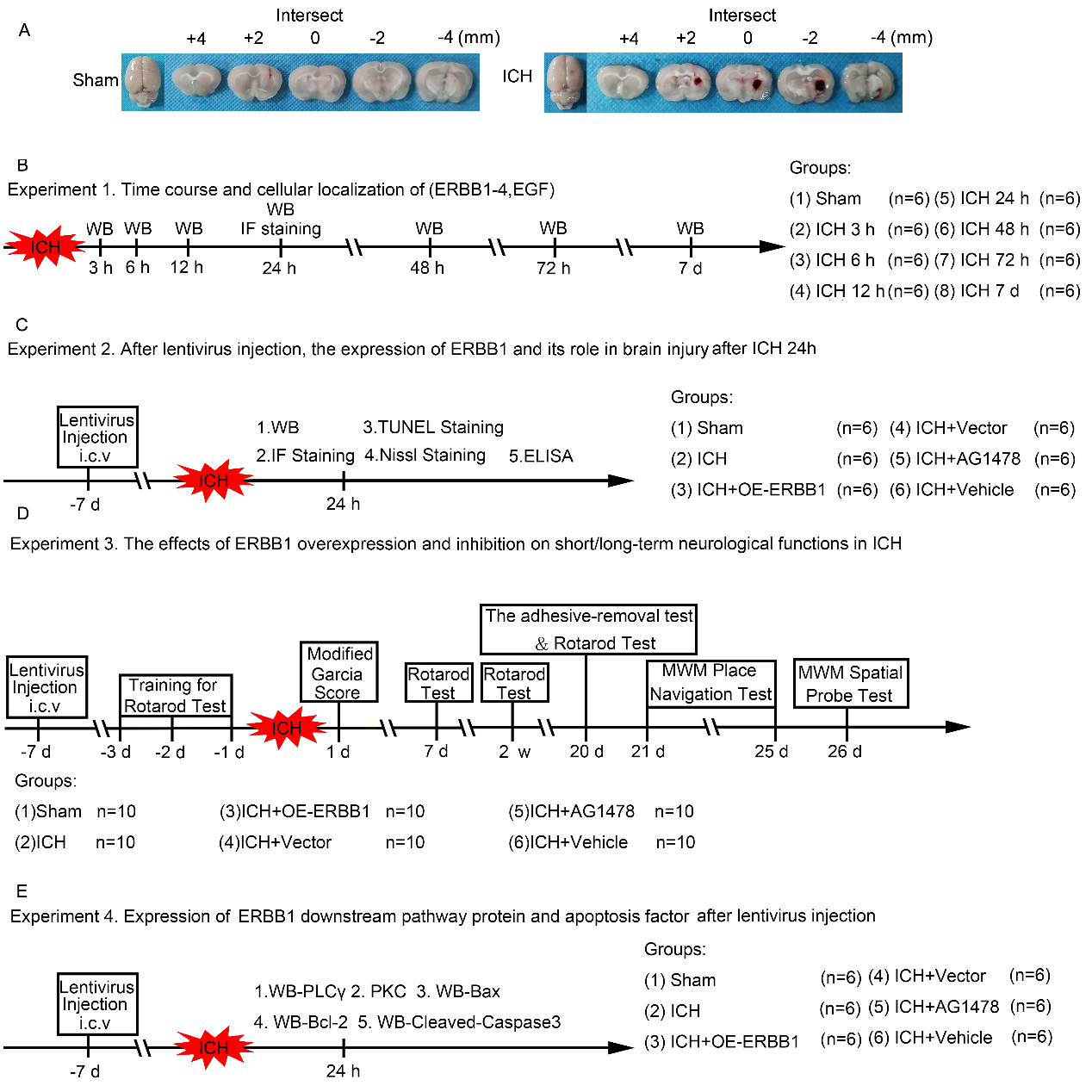


**Figure S1. Experimental design of the time schedule and rat groups.** ICH, intracerebral hemorrhage; WB, western blot; IF, immunofluorescent; Vector, Negative control lentivirus; Vehicle, dimethyl sulfoxide; MWM, Morris water maze. The samples of Experiment 4 and Experiment 2 were shared.


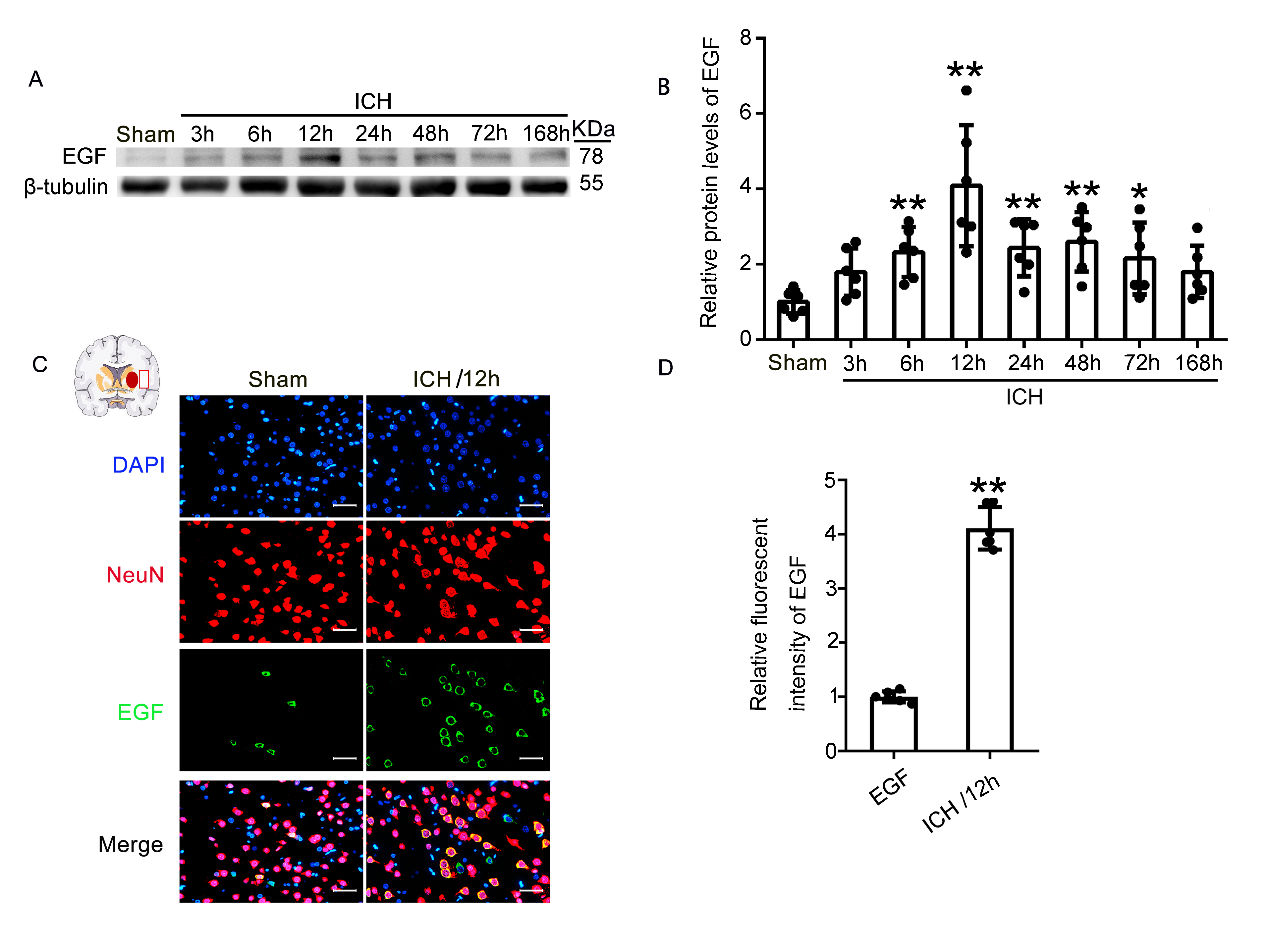


**Figure S2. EGF protein level in brain tissue was increased after ICH modeling.** (A, B). Western blot analysis and quantification of EGF levels at 3, 6, 12, 24, 48, 72 and 168 h after ICH; ∗∗*p* < 0.01 vs. Sham group, ∗*p* < 0.05 vs. Sham group, n = 6. (C, D) Double immunofluorescence staining was performed with EGF antibody (green) and NeuN antibody (red) in brain sections of rats in Sham and ICH 12 h groups. Nuclei were fluorescently labeled with DAPI (blue). Scale bar = 50um, ∗*p* < 0.05 vs. Sham group. All data are displayed as means ± SEM, mean values for Sham group are normalized to 1.0; n = 6, the number of treated animals indicated by “N” in differential experiments.


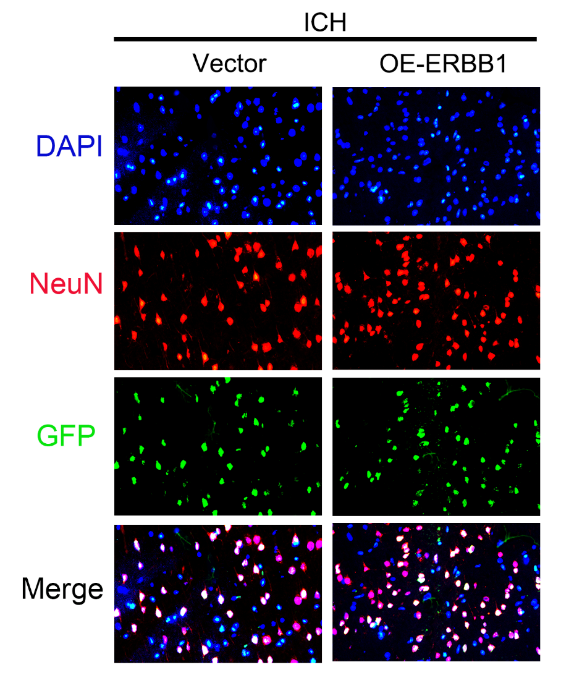


**Figure. S3 The lentivirus transfection efficiency of each group.** Representative microphotographs of IF staining of GFP (green) and neuron (NeuN, red) in the two lentivirus transfection groups were shown.
