# Supplementary material for: ERBB1 alleviates secondary brain injury induced by experimental intracerebral hemorrhage in rats by modulating neuronal death via PLC‐γ/PKC pathway
Source: CNS Neurosci Ther. 2024 Mar 26;30(3):e14679. doi: 10.1111/cns.14679 (PMC10964039; doi:10.1111/cns.14679)
Supplement: Supplementary file 2 — Table S1 [file CNS-30-e14679-s003.docx]

| **Table S1. Summary of experiment groups and mortality rate in the study.** | | | | |
| --- | --- | --- | --- | --- |
| Experiment Groups | Rats used | Successful inclusion | Mortality | Mortality rate (%) |
| **Experiment 1** |  |  |  |  |
| Sham | 6 | 6 | 0 | 0.00 |
| ICH (3h, 6h, 12h, 24h, 48h, 72h, 168h) | 46 | 36 | 10 | 21.73 |
| **Experiment 2, 3 and 4** |  |  |  |  |
| Sham | 16 | 16 | 0 | 0.00 |
| ICH | 19 | 16 | 3 | 15.78 |
| ICH+Vehicle | 19 | 16 | 3 | 15.78 |
| ICH+AG1478 | 21 | 16 | 5 | 23.81 |
| ICH+Vector | 20 | 16 | 4 | 20.00 |
| ICH+OE-ERBB1 | 18 | 16 | 2 | 11.11 |
| **Sham in total** | 22 |  | 0 | 0.00 |
| **ICH in total** | 143 | 116 | 27 | 18.88 |

**Table S2: Detailed statistical table**

| Figures | Description | Test used | Detail value | One- or two- tailed P value? |
| --- | --- | --- | --- | --- |
| Figure 1A | Relative protein level of ERBB1 | One-way ANOVA | Sham vs. ICH 12h: Mean Diff = 0.8833 , 95% CI=0.01715 to 1.750, P=0.0464.  Sham vs. ICH 24h: Mean Diff = 1.990 , 95% CI=1.095 to 2.885, P=0.0006.  Sham vs. ICH 48h: Mean Diff =0.8206 , 95% CI=0.1125 to 1.529, P=0.0273.  Sham vs. ICH 72h: Mean Diff = 0.8794, 95% CI=0.2315 to 1.527, P=0.0128. | Two-tailed |
| Figure 1D | Relative protein level of ERBB4 | One-way ANOVA | Sham vs. ICH 6h: Mean Diff = -0.5047, 95% CI=-0.9268 to -0.08264, P=0.0237.  Sham vs. ICH 12h: Mean Diff = -0.4640 , 95% CI=-0.8926 to -0.03550, P=0.0365. | Two-tailed |
| Figure S2B | Relative protein level of EGF | One-way ANOVA | Sham vs. ICH 6h: Mean Diff =1.325, 95% CI=0.6576 to 1.993, P=0.0013.  Sham vs. ICH 12h: Mean Diff = 3.086, 95% CI=1.594 to 4.577, P=0.0010.  Sham vs. ICH 24h: Mean Diff = 1.438, 95% CI=0.6980 to 2.178, P=0.0015.  Sham vs. ICH 48h: Mean Diff =1.600 , 95% CI=0.8277 to 2.372, P=0.0010.  Sham vs. ICH 72h: Mean Diff = 1.157, 95% CI=0.2449 to 2.068, P=0.0179. | Two-tailed |
| Figure S2D | Relative fluorescent intensity level of EGF | Unpaired t test | Sham vs. ICH 72h: Mean Diff =3.110, 95% CI=2.742 to 3.477, P<0.0001. | Two-tailed |
| Figure 2A | Relative protein level of ERBB1 | One-way ANOVA | Sham vs. ICH: Mean Diff =2.202, 95% CI=1.684 to 2.719, P<0.0001.  ICH+Vehicle vs. ICH+AG1478: Mean Diff =-0.7666, 95% CI=-1.259 to -0.2742, P=0.0060.  ICH+Vetor vs. ICH+OE-ERBB1: Mean Diff =1.756, 95% CI=1.219 to 2.293, P<0.0001. | Two-tailed |
| Figure 2B | Relative protein level of P-ERBB1 | One-way ANOVA | Sham vs. ICH: Mean Diff =0.9446, 95% CI=0.4553 to 1.434, P=0.0016.  ICH+Vehicle vs. ICH+AG1478: Mean Diff =-1.077, 95% CI=-1.994 to -0.1609, P=0.0256.  ICH+Vetor vs. ICH+OE-ERBB1: Mean Diff =1.184, 95% CI=0.4541 to 1.915, P=0.0047. | Two-tailed |
| Figure 2D | Relative fluorescent intensity level of ERBB1 | One-way ANOVA | Sham vs. ICH: Mean Diff =2.484, 95% CI=1.890 to 3.078, P<0.0001.  ICH+Vehicle vs. ICH+AG1478: Mean Diff =-0.9026, 95% CI=-1.161 to -0.6446, P<0.0001.  ICH+Vetor vs. ICH+OE-ERBB1: Mean Diff =2.887, 95% CI=2.589 to 3.185, P<0.0001. | Two-tailed |
| Figure 3C | Percentage of TUNEL-positive cells (%) | One-way ANOVA | Sham vs. ICH: Mean Diff =13.83, 95% CI=12.77 to 14.90, P<0.0001.  ICH+Vehicle vs. ICH+AG1478: Mean Diff =7.333, 95% CI=5.093 to 9.574, P<0.0001.  ICH+Vetor vs. ICH+OE-ERBB1: Mean Diff =-6.333 , 95% CI=-7.994 to -4.673, P<0.0001. | Two-tailed |
| Figure 3D | Number of NISSL-positive neurons in hippocampus (/mm^2^) | One-way ANOVA | Sham vs. ICH: Mean Diff =289.4, 95% CI=223.8 to 355.0, P<0.0001.  ICH+Vehicle vs. ICH+AG1478: Mean Diff =127.3, 95% CI=61.71 to 192.9, P<0.0001.  ICH+Vetor vs. ICH+OE-ERBB1: Mean Diff =-150.5, 95% CI=-216.1 to -84.86, P<0.0001. | Two-tailed |
| Figure 3E | Number of NISSL-positive neurons in cortex(/mm^2^) | One-way ANOVA | Sham vs. ICH: Mean Diff =212.2, 95% CI=140.1 to 284.3, P<0.0001.  ICH+Vehicle vs. ICH+AG1478: Mean Diff =148.5, 95% CI=76.42 to 220.6, P<0.0001.  ICH+Vetor vs. ICH+OE-ERBB1: Mean Diff =-88.73, 95% CI=-160.8 to -16.62, P=0.0103. | Two-tailed |
| Figure 4A | The concentrations of IL-1β in serum(pg/ml) | One-way ANOVA | Sham vs. ICH: Mean Diff =17.34, 95% CI=15.42 to 19.25, P<0.0001.  ICH+Vehicle vs. ICH+AG1478: Mean Diff =12.88, 95% CI=9.398 to 16.36, P<0.0001.  ICH+Vetor vs. ICH+OE-ERBB1: Mean Diff =-10.04, 95% CI=-12.33 to -7.744, P<0.0001. | Two-tailed |
| Figure 4B | The concentrations of IL-1β in CSF(pg/ml) | One-way ANOVA | Sham vs. ICH: Mean Diff =27.45, 95% CI=22.99 to 31.92, P<0.0001.  ICH+Vehicle vs. ICH+AG1478: Mean Diff =9.758, 95% CI=4.996 to 14.52, P=0.0010.  ICH+Vetor vs. ICH+OE-ERBB1: Mean Diff =-17.83, 95% CI=-21.92 to -13.74, P<0.0001. | Two-tailed |
| Figure 4C | The concentrations of TNF-α in serum(pg/ml) | One-way ANOVA | Sham vs. ICH: Mean Diff =201.5, 95% CI=178.2 to 224.8, P<0.0001.  ICH+Vehicle vs. ICH+AG1478: Mean Diff =94.39, 95% CI=61.78 to 127.0, P<0.0001.  ICH+Vetor vs. ICH+OE-ERBB1: Mean Diff =-134.7, 95% CI=-161.1 to -108.2, P<0.0001. | Two-tailed |
| Figure 4D | The concentrations of TNF-α in CSF(pg/ml) | One-way ANOVA | Sham vs. ICH: Mean Diff =222.9, 95% CI=193.8 to 252.0, P<0.0001.  ICH+Vehicle vs. ICH+AG1478: Mean Diff =121.6, 95% CI=83.24 to 160.0, P<0.0001.  ICH+Vetor vs. ICH+OE-ERBB1: Mean Diff =-115.9, 95% CI=-146.9 to -84.88, P<0.0001. | Two-tailed |
| Figure 4E | The concentrations of LDH in CSF | One-way ANOVA | Sham vs. ICH: Mean Diff =37.77, 95% CI=32.91 to 42.62, P<0.0001.  ICH+Vehicle vs. ICH+AG1478: Mean Diff =97.76, 95% CI=74.40 to 121.1, P<0.0001.  ICH+Vetor vs. ICH+OE-ERBB1: Mean Diff =-15.27, 95% CI=-20.90 to -9.645, P=0.0001. | Two-tailed |
| Figure 4F | The ROS levels in brain tissues | One-way ANOVA | Sham vs. ICH: Mean Diff =1.766, 95% CI=0.9437 to 2.589, P=0.0007.  ICH+Vehicle vs. ICH+AG1478: Mean Diff =1.141, 95% CI=0.02178 to 2.261, P=0.0465.  ICH+Vetor vs. ICH+OE-ERBB1: Mean Diff =-0.9215, 95% CI=-1.620 to -0.2233, P=0.0148. | Two-tailed |
| Figure 4G | Modified Garcia score | One-way ANOVA | Sham vs. ICH: Mean Diff =7.100, 95% CI=5.899 to 8.301, P<0.0001.  ICH+Vehicle vs. ICH+AG1478: Mean Diff =3.600, 95% CI=2.399 to 4.801, P<0.0001.  ICH+Vetor vs. ICH+OE-ERBB1: Mean Diff =-3.700, 95% CI=-4.901 to -2.499, P<0.0001. | Two-tailed |
| Figure 4H | Rotarod time (s) | Two-way ANOVA | Sham vs. ICH: Mean Diff =21.84, 95% CI=19.49 to 24.19, P<0.0001.  ICH+Vehicle vs. ICH+AG1478: Mean Diff =4.781, 95% CI=2.431 to 7.131, P<0.0001.  ICH+Vetor vs. ICH+OE-ERBB1: Mean Diff =-6.471, 95% CI=-8.821 to -4.121, P<0.0001. | Two-tailed |
| Figure 4I | Adhesive-removal time (s) | Two-way ANOVA | Sham vs. ICH: Mean Diff =-43.78, 95% CI=-47.51 to -40.05, P<0.0001.  ICH+Vehicle vs. ICH+AG1478: Mean Diff =-11.51, 95% CI=-15.24 to -7.777, P<0.0001.  ICH+Vetor vs. ICH+OE-ERBB1: Mean Diff =11.37, 95% CI=7.640 to 15.11, P<0.0001. | Two-tailed |
| Figure 5B | Average swimming speed (cm/s) | One-way ANOVA | Sham vs. ICH: Mean Diff =-0.4494, 95% CI=-3.496 to 2.597, P=0.9974.  ICH+Vehicle vs. ICH+AG1478: Mean Diff =0.1870, 95% CI=-2.860 to 3.234, P=0.9999.  ICH+Vetor vs. ICH+OE-ERBB1: Mean Diff =0.8260, 95% CI=-2.221 to 3.873, P=0.9596. | Two-tailed |
| Figure 5C | Morris water maze latency (s) | Two-way ANOVA | Sham vs. ICH: Mean Diff =-11.00, 95% CI=-18.89 to -3.112, P=0.0014.  ICH+Vehicle vs. ICH+AG1478: Mean Diff =-8.864, 95% CI=-16.98 to -0.7450, P=0.0239.  ICH+Vetor vs. ICH+OE-ERBB1: Mean Diff =6.227, 95% CI=-1.757 to 14.21, P<0.0001. | Two-tailed |
| Figure 5D | Swimming distance (cm) | Two-way ANOVA | Sham vs. ICH: Mean Diff =-334.1, 95% CI=-436.9 to -231.3, P<0.0001.  ICH+Vehicle vs. ICH+AG1478: Mean Diff =-258.4, 95% CI=-361.2 to -155.6, P<0.0001.  ICH+Vetor vs. ICH+OE-ERBB1: Mean Diff =209.2, 95% CI=106.3 to 312.0, P<0.0001. | Two-tailed |
| Figure 5F | Time in target platform (%) | One-way ANOVA | Sham vs. ICH: Mean Diff =0.1116, 95% CI=0.05649 to 0.1667, P<0.0001.  ICH+Vehicle vs. ICH+AG1478: Mean Diff =0.06780, 95% CI=0.01269 to 0.1229, P=0.0092.  ICH+Vetor vs. ICH+OE-ERBB1: Mean Diff =-0.07880, 95% CI=-0.1339 to -0.02369, P=0.0018. | Two-tailed |
| Figure 6A | Relative protein level of EGF | One-way ANOVA | Sham vs. ICH: Mean Diff =2.928, 95% CI=2.601 to 3.255, P<0.0001.  ICH+Vehicle vs. ICH+AG1478: Mean Diff =-0.1067, 95% CI=-0.6150 to 0.4016, P=0.6501.  ICH+Vetor vs. ICH+OE-ERBB1: Mean Diff =0.3741, 95% CI=-0.1641 to 0.9123, P=0.1525. | Two-tailed |
| Figure 6B | Relative protein level of PLC γ | One-way ANOVA | Sham vs. ICH: Mean Diff =1.646, 95% C=I1.063 to 2.229, P<0.0001.  ICH+Vehicle vs. ICH+AG1478: Mean Diff =-1.423, 95% CI=-2.371 to -0.4741, P=0.0075.  ICH+Vetor vs. ICH+OE-ERBB1: Mean Diff =2.652, 95% CI=1.369 to 3.935, P=0.0010. | Two-tailed |
| Figure 6C | Relative protein level of PKC | One-way ANOVA | Sham vs. ICH: Mean Diff =1.653, 95% CI=0.9511 to 2.355, P=0.0004.  ICH+Vehicle vs. ICH+AG1478: Mean Diff =-1.795, 95% CI=-2.549 to -1.041, P=0.0003.  ICH+Vetor vs. ICH+OE-ERBB1: Mean Diff =1.943, 95% CI=0.3143 to 3.571, P=0.0240. | Two-tailed |
| Figure 6D | Relative protein level of Bax | One-way ANOVA | Sham vs. ICH: Mean Diff =1.052, 95% CI=0.3146 to 1.790, P=0.0098.  ICH+Vehicle vs. ICH+AG1478: Mean Diff =1.012, 95% CI=0.03948 to 1.985, P=0.0429.  ICH+Vetor vs. ICH+OE-ERBB1: Mean Diff =-0.8347, 95% CI=-1.398 to -0.2715, P=0.0080. | Two-tailed |
| Figure 6E | Relative protein level of Bcl-2 | One-way ANOVA | Sham vs. ICH: Mean Diff =-0.4613, 95% CI=-0.7712 to -0.1513, P=0.0078.  ICH+Vehicle vs. ICH+AG1478: Mean Diff =-0.4031, 95% CI=-0.7785 to -0.02775, P=0.0378.  ICH+Vetor vs. ICH+OE-ERBB1: Mean Diff =0.6009, 95% CI=0.09366 to 1.108, P=0.0248. | Two-tailed |
| Figure 6F | Relative protein level of cleaved caspase-3 | One-way ANOVA | Sham vs. ICH: Mean Diff =1.470, 95% CI=0.9179 to 2.022, P=0.0001.  ICH+Vehicle vs. ICH+AG1478: Mean Diff =2.529, 95% CI=1.361 to 3.697, P=0.0007.  ICH+Vetor vs. ICH+OE-ERBB1: Mean Diff =-1.329, 95% CI=-1.944 to -0.7145, P=0.0007. | Two-tailed |


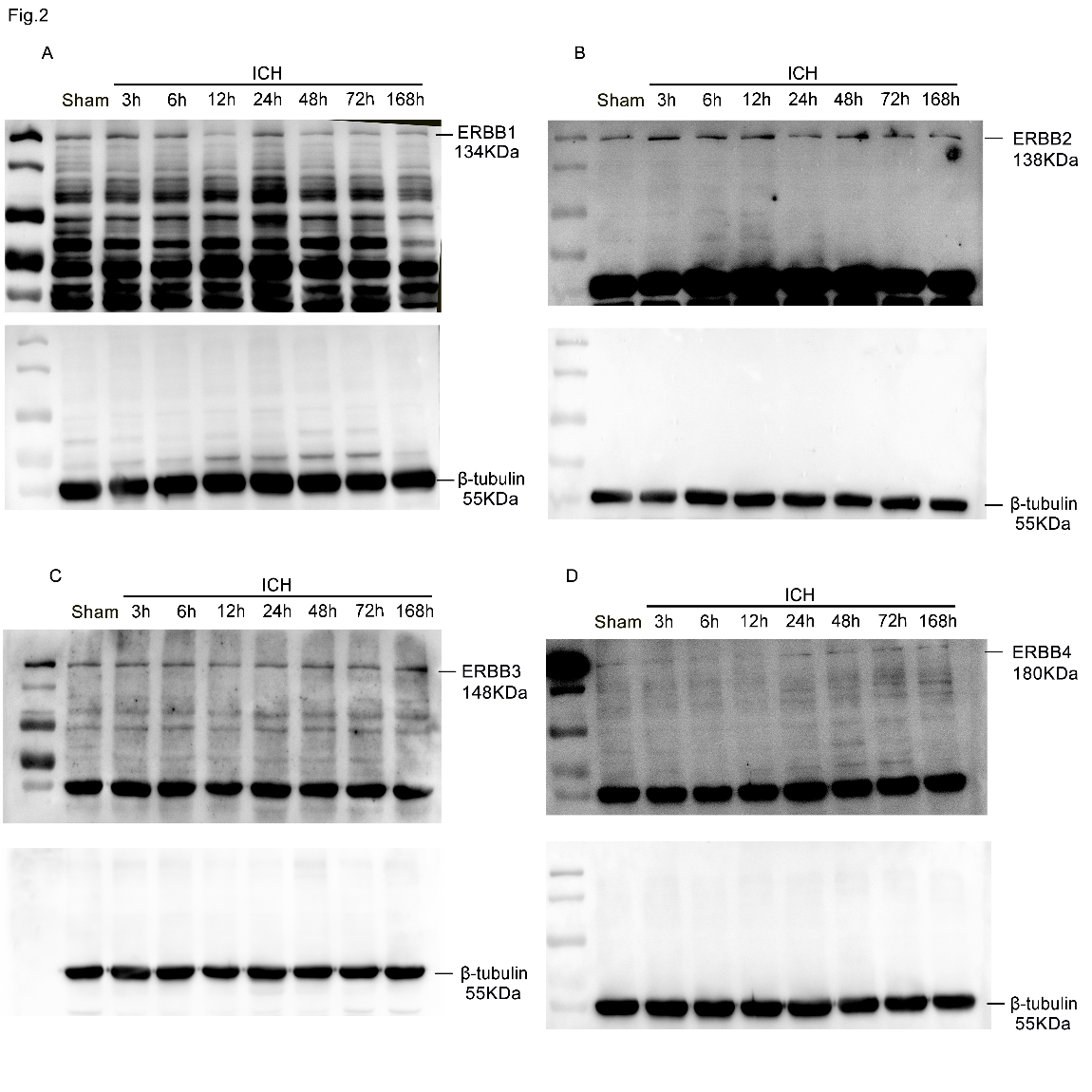


Fig. S1 Images of full-length western blots presented in Figure 2 of the article.


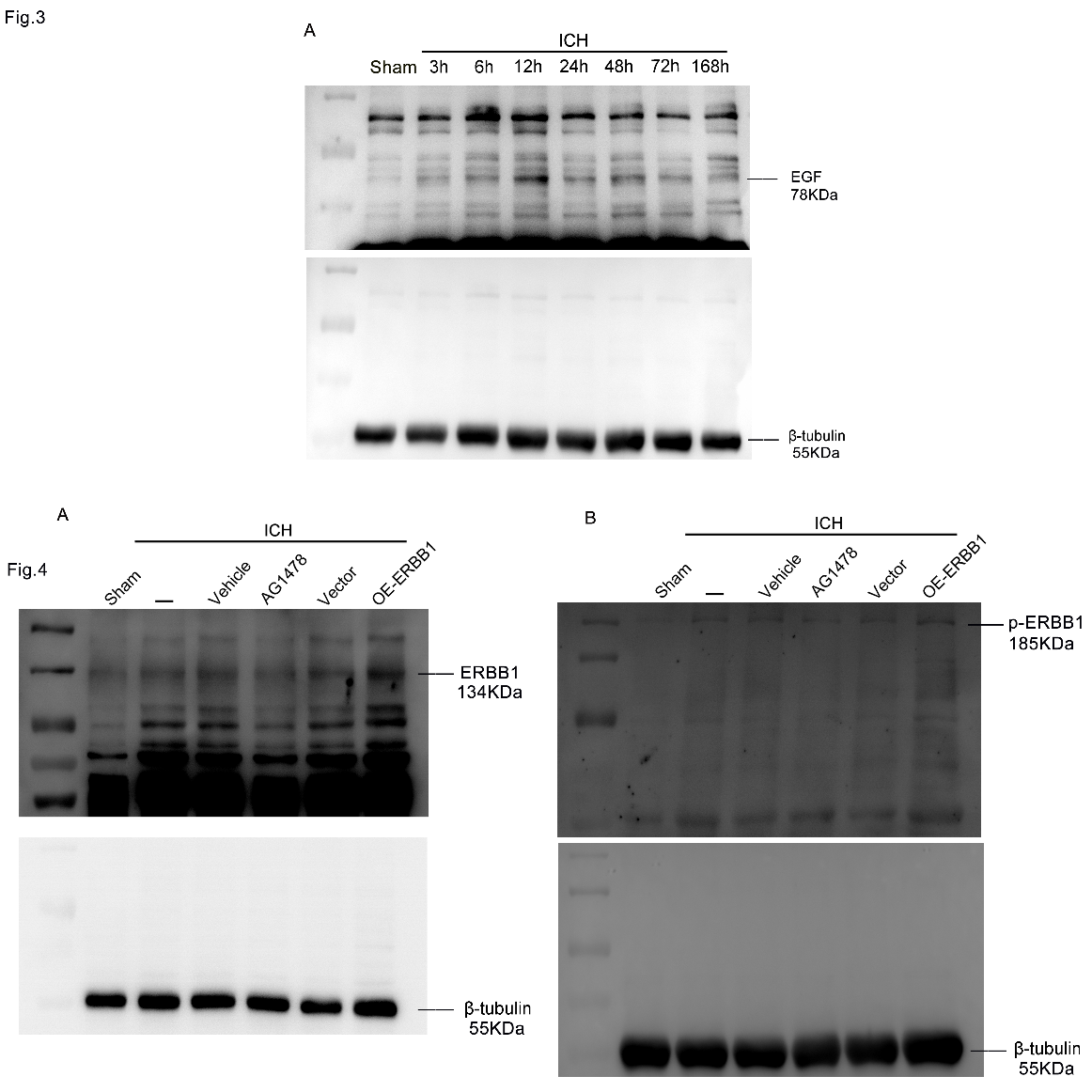


Fig. S2 Images of full-length western blots presented in Figure 3 and 4 of the article.


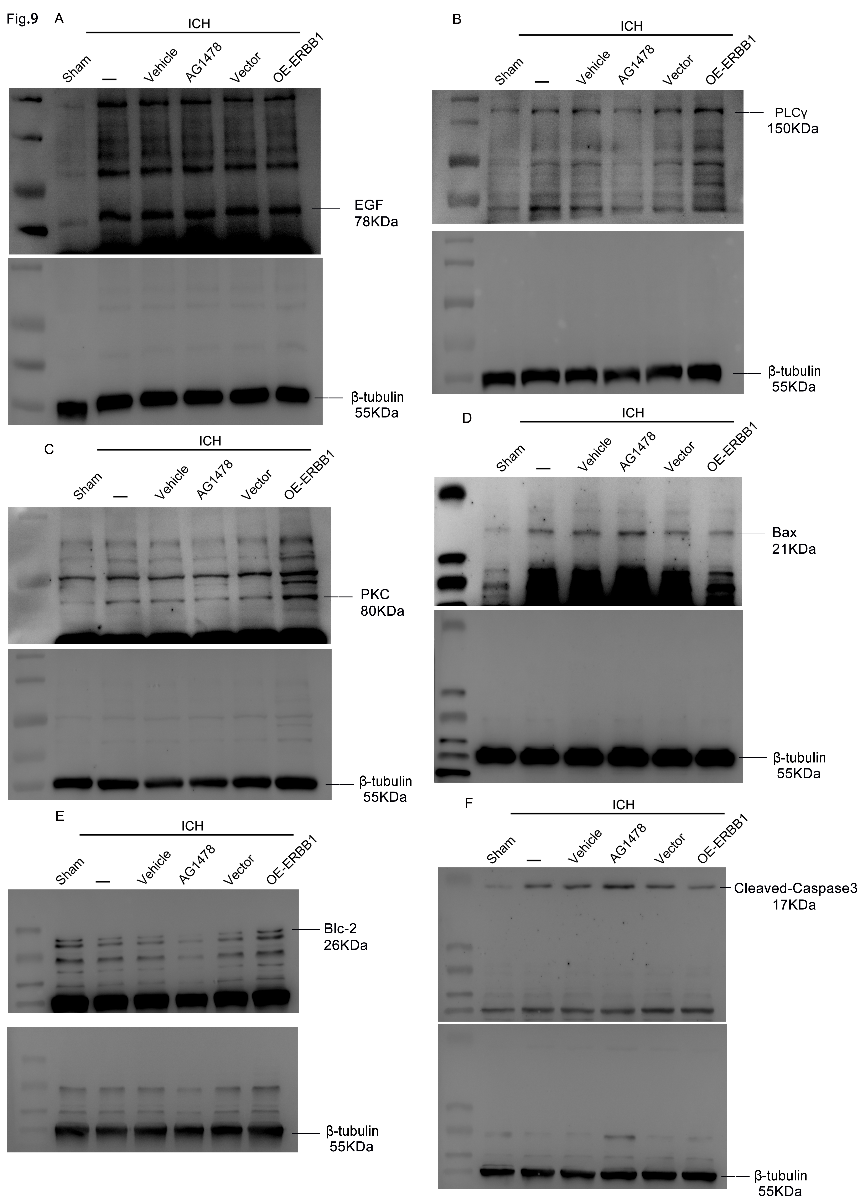


Fig. S3 Images of full-length western blots presented in Figure 9 of the article.
