# Supplementary material for: ERBB1 alleviates secondary brain injury induced by experimental intracerebral hemorrhage in rats by modulating neuronal death via PLC‐γ/PKC pathway
Source: CNS Neurosci Ther. 2024 Mar 26;30(3):e14679. doi: 10.1111/cns.14679 (PMC10964039; doi:10.1111/cns.14679)

Full unedited gel/blot for Figure 1

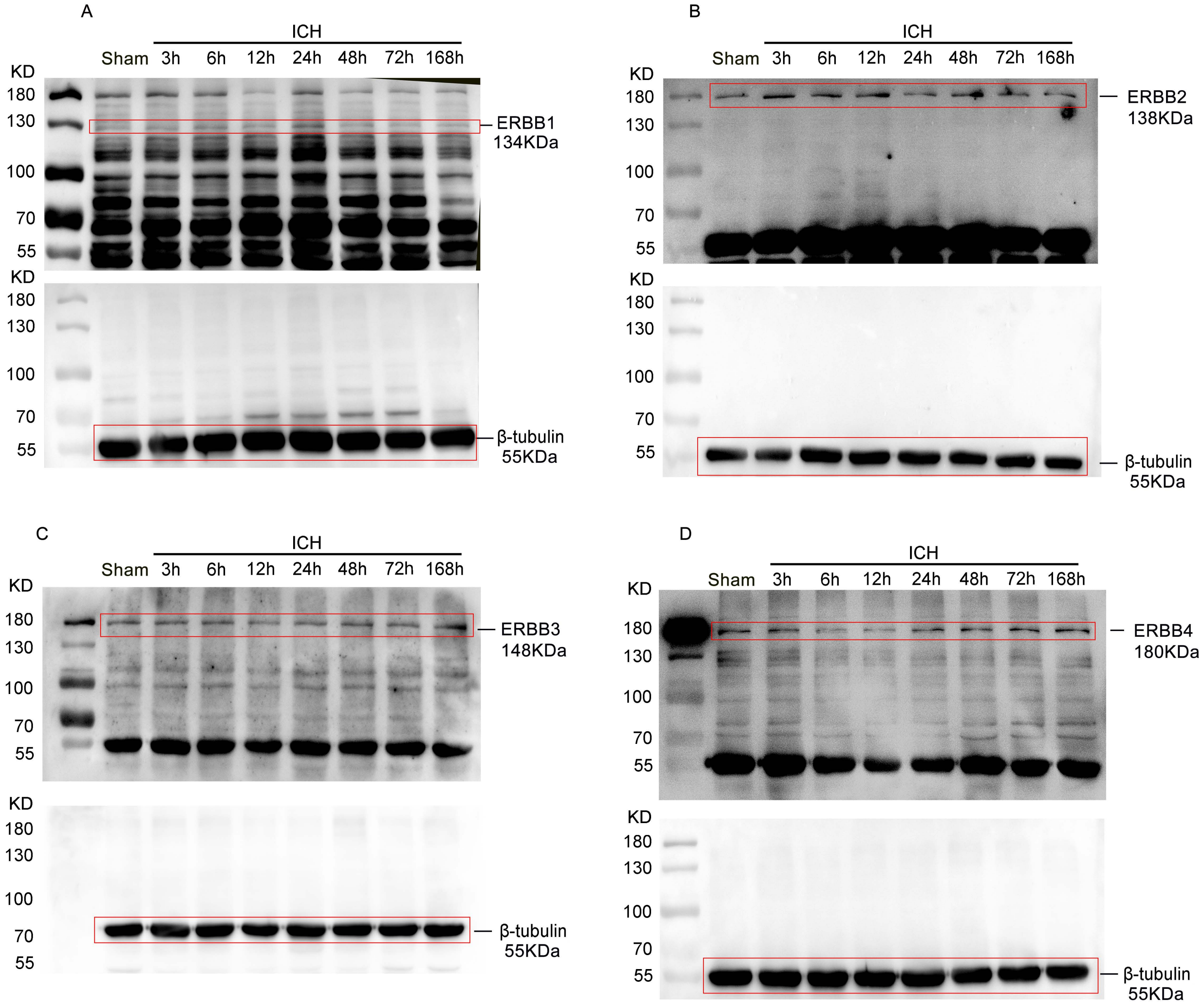

Full unedited gel/blot for Figure 2

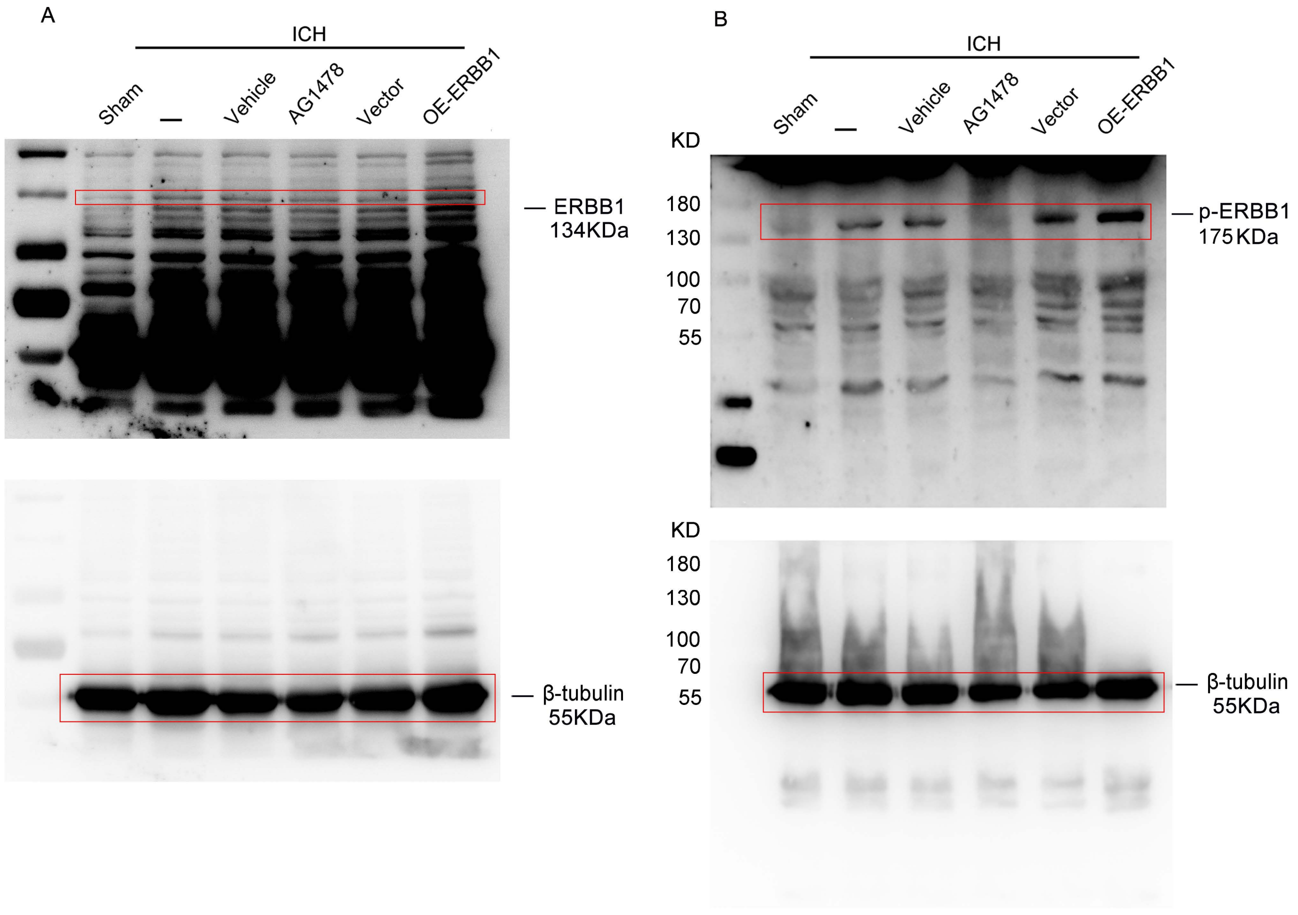

Full unedited gel/blot for Figure 6

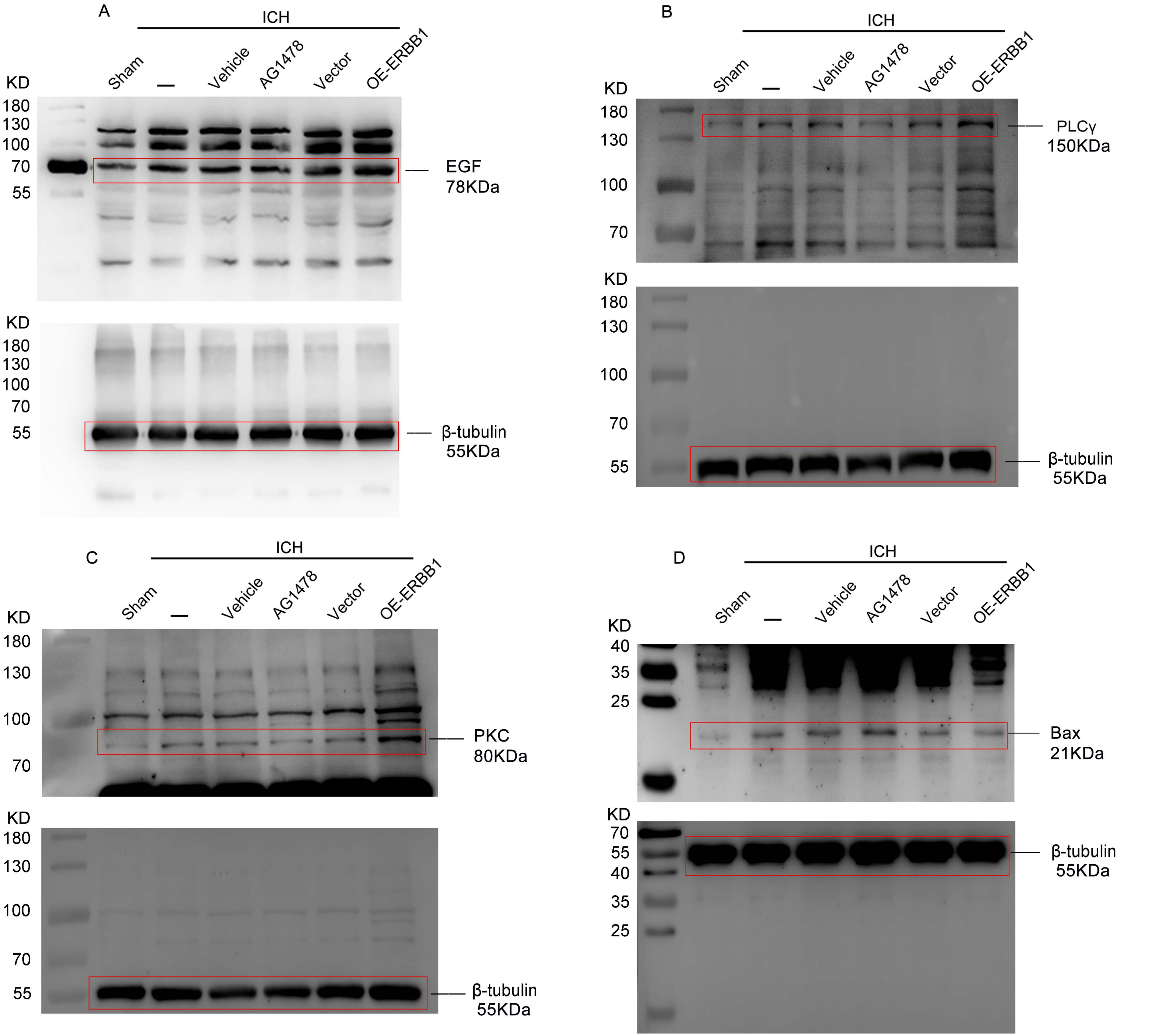

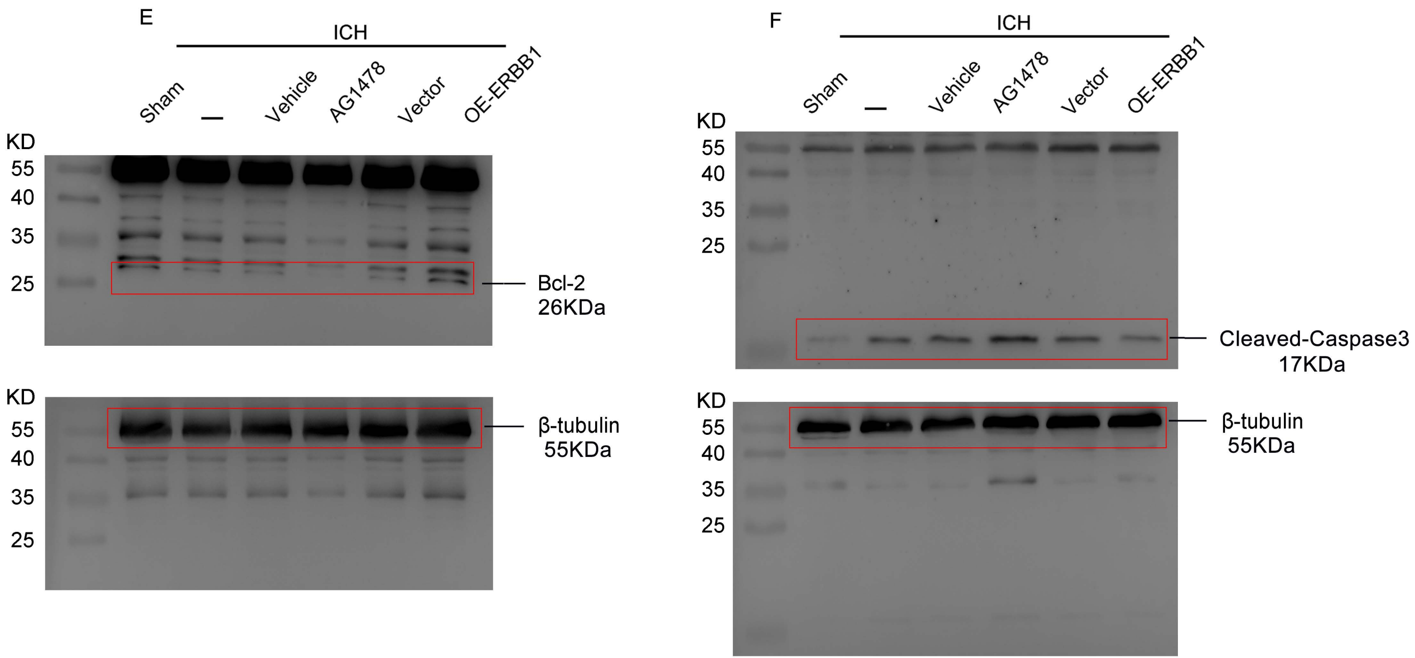

E
ICH
Sham
—
Vehicle
AG1478
Vector
OE-ERBB1
KD
55
40
35
25
Bcl-2
26KDa
KD
55
40
35
25
β-tubulin
55KDa
F
ICH
Sham
—
Vehicle
AG1478
Vector
OE-ERBB1
KD
55
40
35
25
Cleaved-Caspase3
17KDa
KD
55
40
35
25
β-tubulin
55KDa

Full unedited gel/blot for Figure S2

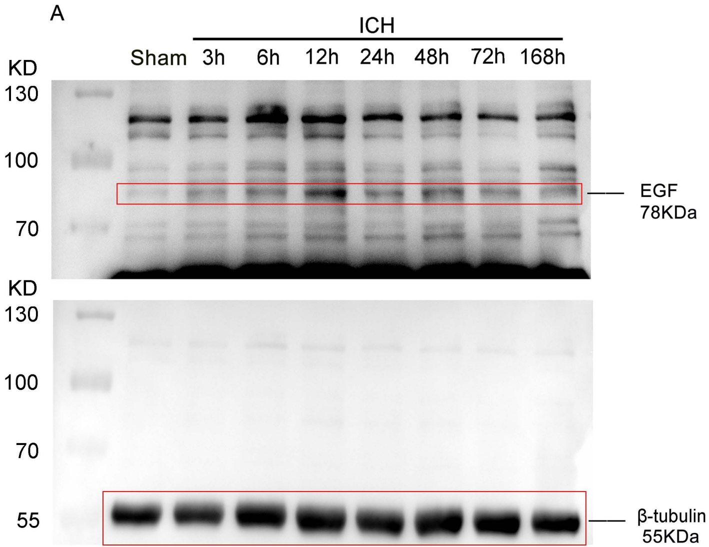

Supplement: Supplementary file 3 — Appendix S2 [file CNS-30-e14679-s001.pdf]
